# A new natural Cyperol A together with five known compounds from *Cyperus rotundus* L.: isolation, structure elucidation, DFT analysis, insecticidal and enzyme-inhibition activities and *in silico* study†

**DOI:** 10.1039/d5ra00505a

**Published:** 2025-04-11

**Authors:** Saqib Hussain Bangash, Muhammad Ibrahim, Akbar Ali, Chen-Yang Wei, Amjad Hussain, Moazama Riaz, Muhammad Fayyaz Ur Rehman, Faiz Ahmed, Rashad Al-Salahi, Wen-Wei Tang

**Affiliations:** a Guangxi Key Laboratory of Agro-Environment and Agric-Product Safety, National Demonstration Center for Experimental Plant Science Education, College of Agriculture, Guangxi University Nanning Guangxi People's Replublic of China wenweitg@163.com; b Department of Applied Chemistry, Government College University Faisalabad Pakistan ibrahimchem@gmail.com; c Department of Chemistry, Government College University Faisalabad Pakistan akbarali@gcuf.edu.pk akbarchm@gmail.com; d Institute of Chemistry, University of Okara Okara-56300 Punjab Pakistan; e Institute of Chemistry, University of Sargodha Sargodha Pakistan; f Department of Pharmaceutical Chemistry, College of Pharmacy, King Saud University Riyadh 11451 Saudi Arabia

## Abstract

One new natural benzaldehyde derivative (1), together with five known compounds, was isolated from the methanolic extract of the whole plant of *Cyperus rotundus* L., which is a globally distributed noxious weed. The structure of compound (1) (named Cyperol A) was determined using various NMR methods, including ^1^H, ^13^C, COSY, HMBC, HSQC and NOESY, and mass spectrometric techniques, including EIMS. The newly isolated compound (1) was subjected to optimization using computer-assisted calculation *via* DFT methods for natural bond orbital (NBO) and frontier molecular orbital (FMO) analyses and compared with carbofuran, which is used to control the pest brown planthopper. The *in vitro* insecticidal efficacy of compounds 1–6 was evaluated against *Nilaparvata lugens*. Compound 1 demonstrated exceptional lethal and notable enzyme inhibitory effects. Furthermore, compound 1 was investigated *in silico* for its anti-pesticidal activities targeting the BPH (*Nilaparvata lugens* (Stål)) key enzymes, such as glutathione S-transferase (GST) and acetylcholinesterase (AChE). Compound 1 showed good docking scores of −9.75 kcal mol^−1^ against GST, forming hydrogen bonds with its active site, and −10.56 kcal mol^−1^ with AChE owing to its high potential for hydrogen bonding.

## Abstract 2

One new natural benzaldehyde derivative (1), together with five known compounds, was isolated from the methanolic extract of the whole plant of *Cyperus rotundus* L., which is a globally distributed noxious weed.

## Graphical abstract

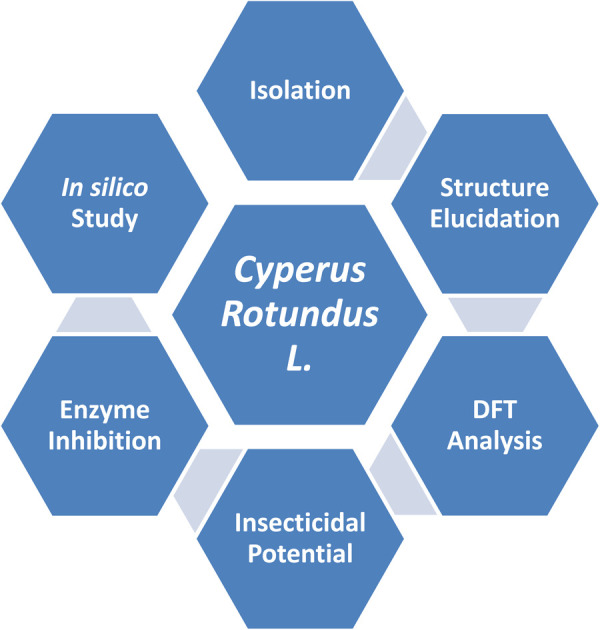

## 1. Introduction

Purple nutsedge (*Cyperus rotundus* L.) is a member of the Cyperaceae family and is considered one of the most noxious weeds in the world.^1^ It is widely distributed in the tropical and subtropical regions, infesting fields that include sugarcane, maize, and even rice. In the rice fields, brown planthoppers (*Nilaparvata lugens* (Stål)) have been reported as one of the most common pests. Interestingly, *C. rotundus* has never been reported as a food source or host of brown planthoppers.^2^ Meanwhile, studies have shown that this weed contains many phytochemicals, including steroids, triterpenes, tannins, anthraquinones, and alkaloids, which are known for their potent insecticidal activities.^3^ Additionally, oils extracted from this weed have been reported to function as natural insecticides, serving as toxic fumigants^4^ and insect repellents^5^ with ovicidal potential. Thus, in recent years, there has been a growing interest in using compounds derived from *C. rotundus* to manage pests, particularly in the stored products.^6^ Therefore, we speculate that certain natural phytochemicals may be responsible for the brown planthoppers not choosing *C. rotundus* as a food source or host.

Density functional theory (DFT) is an exciting computer-assisted calculation technique used to compute molecular structures, which aids in identifying plant-derived substances and confirming their vibrational frequencies and molecular parameters derived from experimental spectroscopic techniques. Additionally, it plays a key role in facilitating molecular modelling, which has revolutionized our understanding of the fundamental physical and chemical interactions and the prediction of the physiochemical properties of natural compounds extracted from plants.^7–9^ The toxicity of insecticidal compounds is often linked to their impact on biochemical pathways, particularly through enzyme inhibition activities.^10^ Organophosphates, carbamates, and certain botanical compounds inhibit acetylcholinesterase (AChE) activity, leading to neuronal overstimulation, muscle tremors, convulsions, and ultimately insect mortality.^11,12^ Furthermore, molecular docking is an emerging and powerful technique that enables in-depth exploration of ligand–receptor interactions and precise predictions of optimal binding configurations between ligands and targeted proteins.^13^ These methods efficiently screen large chemical libraries and assist researchers in understanding the extracted natural compounds.^14^

In the current study, five known compounds and Cyperol A (a novel natural compound) were isolated from the whole plant of *C. rotundus*. The structure of Cyperol A was elucidated using spectroscopic techniques, including NMR, EIMS, and HRMS. The newly isolated natural aromatic aldehyde was subjected to DFT studies, along with assessments of its lethal activity and inhibitory effects on AChE and GST against *N. lugens*. Additionally, molecular docking studies were conducted to evaluate its potential as a natural insecticide. Notably, the results from these analyses provide valuable insights into *C. rotundus* as a promising source of eco-friendly insecticides that can contribute to sustainable pest management solutions in agriculture.

## 2. Results and discussion

**Fig. 1:**
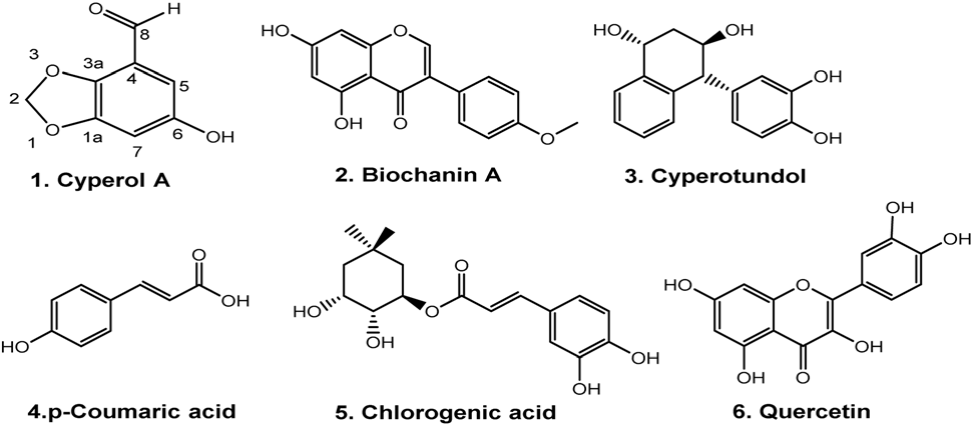
Structures of isolated compounds 1–6.

The methanolic extract (300 g) of the whole plant of *C. rotundus* was fractionated using repeated column chromatography over silica gel, Sephadex (LH-20) and normal phase chromatography, resulting in the isolation of a new natural product (1), along with five known compounds: biochanin A (2), cyperotundol (3), *p*-coumaric acid (4), chlorogenic acid (5) and quercetin (6)^15,16^ (Fig. 1).

**Fig. 2:**
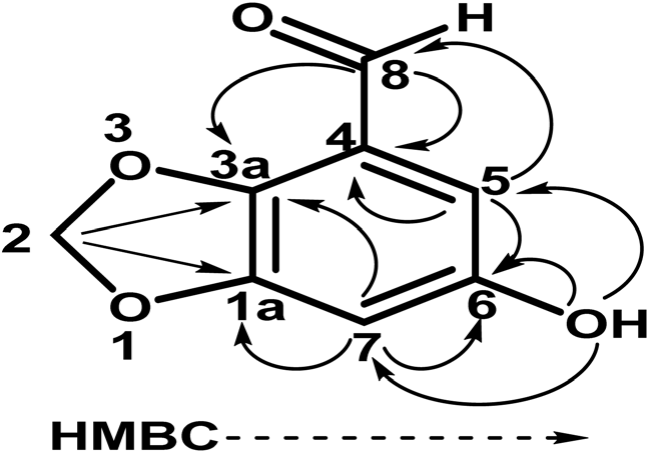
Key HMBC correlations in Cyperol A.

Compound 1 was isolated as a yellowish amorphous solid; m.p: 186–188 °C, IR (CHCl_3_) *ύ*_max_ cm^−1^: 3400 cm^−1^ (OH), 1720 cm^−1^ (C=O); UV *λ*_max_ nm (MeOH) (log *ε*): 305 (2.8); EI-MS: *m*/*z* [M]^+^ = 166.13 (calculated 166.13 for C_8_H_6_O_4_). The ^1^H-NMR spectrum of compound 1 was resolved into four singlets including one signal in the downfield region resonating at *δ*_H_ 10.21 (H-8), characteristic of an aldehydic proton, which indicated the presence of an aldehydic group, and two aromatic methine protons at *δ*_H_ 7.24 (1H, s, H-5) and *δ*_H_ 7.33 (1H, s, H-7) indicated a tetra-substituted aromatic ring in the compound. A doubly integrated singlet, characteristic of methylene protons, resonated at *δ*_H_ 6.20 (2H, s, H-2), while a singlet corresponding to a single OH group was observed at *δ*_H_ 7.52 (Fig. S1†). The ^13^C-NMR spectrum of compound 1 was resolved into 8 resonances. The DEPT-90 and 135 helped in identifying the presence of one oxy-methylene group, indicated by a downfield signal at *δ*_C_ 104.0 (C-2), and two methine carbons at *δ*_C_ 107.7 (C-5) and *δ*_C_ 105.3 (C-7). The four quaternary carbons in the aromatic region were assigned as *δ*_C_ 128.3 (C-4), *δ*_C_ 152.3 (C-6), *δ*_C_ 151.6 (C-1a), and *δ*_C_ 147.3 (C-3a). A single downfield signal at *δ*_C_ 186.9 (C-8) in the broadband, DEPT-90 and DEPT-135 of the ^13^C-NMR spectra indicated the presence of an aldehyde function in the compound. The HMBC spectrum (Fig. 2 and S8†) displayed the interactions of the OH proton (*δ*_OH̲_ 7.52) with C-5 and C-7, which helped in determining the position of the hydroxy group at C-6. Other observed HMBC correlations included H-5 with C-4, C-6, C-7, C-3a and C-8; H-7 with C-5, C-6, C-1a, C-9 on one side; and the HMBC interactions of H-8 with C-4, C-5 and C-3a, which helped in identifying the position of the carboxyl group at C-4 on the aromatic ring. The important HMBC interactions of *δ* 2H at C-2 with C-1a and C-3a confirmed the presence of a dioxole group and its position at C-1a and C-3a on the aromatic ring. These data closely aligned with those of 6-hydroxy-2*H*-1,3-benzodioxole-5-carbaldehyde,^17^ confirming the position of the aldehyde group at C-4 in compound 1. Additionally, the NOSEY and COSY NMR spectra showed no correlations (Fig. S5 and S6,† respectively). Consequently, the structure of compound 1 was elucidated as 6-hydroxy-2*H*-1,3-benzodioxole-4-carbaldehyde, as shown in Fig. 1. Thus, the compound was named Cyperol A.

The spectroscopic data of compounds 2–6 were compared with those of previously reported compounds and were found to be identical to biochanin A (2), cyperotundol (3), *p*-coumaric acid (4), chlorogenic acid (5) and quercetin (6).^15,16^

### 2.1. Computational procedures

#### 2.1.1. DFT calculations

**Fig. 3:**
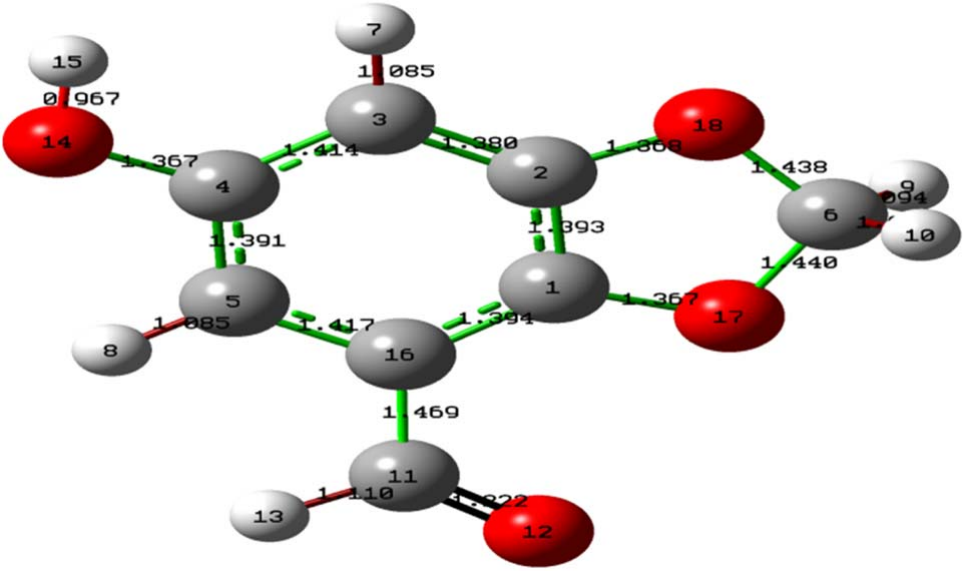
Optimized geometry of Cyperol A using DFT/B3LYP/6-311G (d,p).

The molecular geometry of Cyperol A was fully optimized (Fig. 3) using the B3LYP/6-311G (d,p) DFT method, which enabled the computation of theoretical vibrational spectra and bond lengths.

#### 2.1.2. Natural bond orbital (NBO) analysis

The natural bond orbital (NBO) analysis is used to predict intra- and intermolecular bonding, charge transfer, and hyperconjugation in the molecular orbitals (bonding and antibonding).^18^ This type of molecular orbital interaction is assessed by analyzing the stabilization energy, *E*^(2)^. Its value correlates with the strength of interaction between electron donors and acceptors, and a higher value indicates greater conjugation in the molecular system. This conjugation affects the bond lengths between atoms within the system due to the shifting of electron density, which allows us to predict the stability and reactive sites within the molecule. A second-order perturbation analysis of the Fock matrix was performed to assess such molecular interactions. The stabilization energy, *E*^(2)^, for *i* → *j* delocalization between each donor (*i*) and acceptor (*j*) is calculated as:^19^1
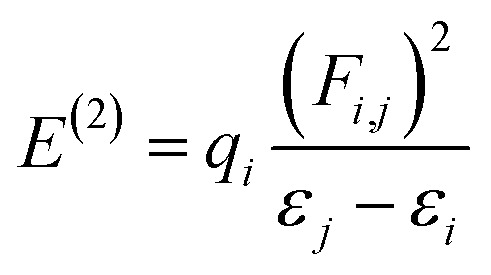
*q*_*i*_ = population of the donor orbital, *ε*_*j*_, *ε*_*i*_ = diagonal elements (orbital energies), *F*(*i*, *j*) = off-diagonal Fock matrix NBO element between *i* and *j* orbital, and *ε*_*j*_ − *ε*_*i*_ = the energy difference between the *i* and *j* NBO orbitals of the donor and acceptor.^20^

In Cyperol A, the highest stabilization energy (*E*^2^) is reported (Table S1†) due to the conjugate interaction of the filled molecular orbital lone pair of (O12), (O17) and (O18) with unfilled (π* C11=C16), (π* C1=C2) and (π* C1=C2) molecular orbital, respectively. The *E*^2^ of these interactions are 20.39, 28.14 and 27.07 kJ mol^−1^, respectively, which is due to intramolecular charge transfer (ICT), resulting in the high stabilization of the investigated compound. The strong electron donor interactions, π(C1=C2) → π*(C3=C4) (18.50 kJ mol^−1^), π(C1=C2) → π*(C5=C16) (18.73 kJ mol^−1^), π(C3=C4) → π*(C1=C2) (17.87 kJ mol^−1^), π(C3=C4) → π*(C5=C16) (15.20 kJ mol^−1^), π(C5=C16) → π*(C1=C2) (18.35 kJ mol^−1^), π(C5=C16) → π*(C3=C4) (15.29 kJ mol^−1^), and π(C5=C16) → π*(C11 = O12) (16.59 kJ mol^−1^) in the aromatic ring are identified. Another prominent intramolecular hyperconjugative interaction occurs between the lone pair on (O12) and the antibonding σ* orbitals of (C11) and σ*(C11–H13) having the *E*^2^ of 11.52 and 19.07 kJ mol^−1^, respectively. These intramolecular charge transfers (ICT) contribute to the overall stabilization of the investigated compound.

#### 2.1.3. Frontier molecular orbital (FMO) analysis

**Fig. 4:**
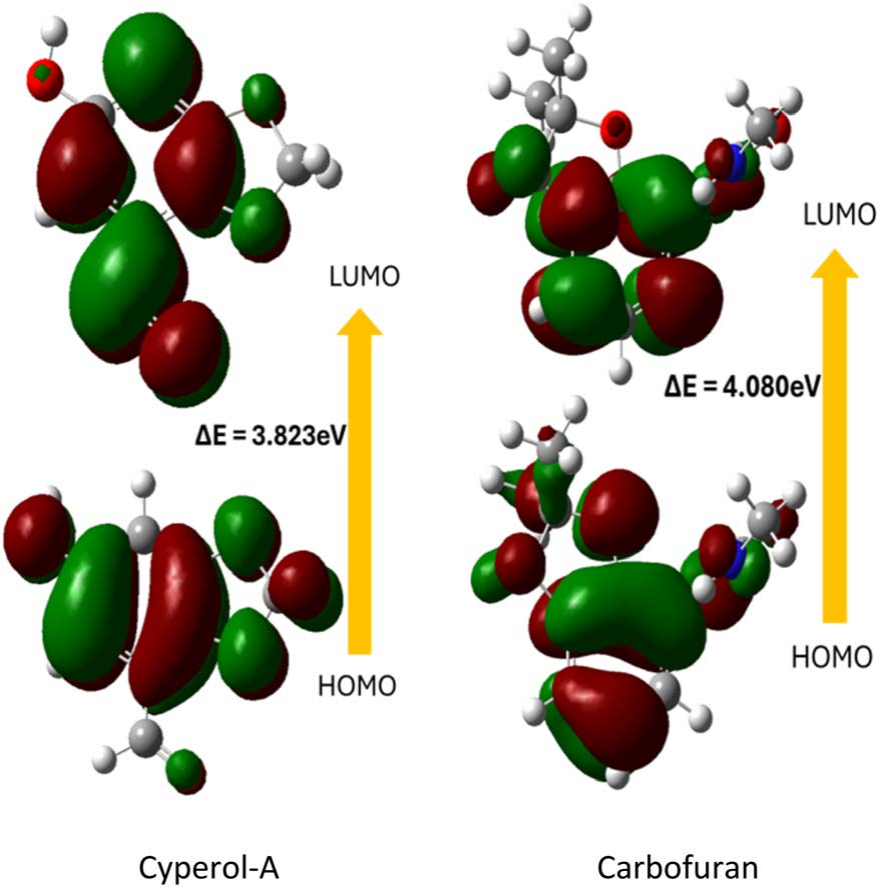
Frontier molecular orbitals of Cyperol A and carbofuran at the B3LYP/6-311G (d,p) level; red and green color represent positive and negative phases, respectively.

Frontier molecular orbitals (FMOs) elucidate molecular interactions and charge transfer in the HOMO (highest occupied molecular orbital) and LUMO (lowest unoccupied molecular orbital).^21^ This approach also assesses chemical reactivity, kinetic stability, and optical polarizability.^22^ The electron distribution from the HOMO to the LUMO was used to calculate the energy gaps (*E*) between the FMOs of compound 1 at the B3LYP/6-311G (d,p) level. The energy difference, Δ*E*, was calculated to be 3.823 eV between the HOMO and LUMO, which is less than that of the standard BPH insecticide carbofuran (Fig. 4). This predicts that it is more biologically active than carbofuran due to the presence of a dioxole ring, which is less stable than furan. The HOMO charge density was saturated on the carbon atoms of the benzene ring that has a hydroxyl group substituent.

Meanwhile, the charges were transferred to the fused carbon atom and the adjacent carbon atom of the benzodioxole nucleus. In FMO analysis, higher reactivity is estimated by a low energy gap, while lower reactivity is estimated by a larger energy gap.^23^ Besides the FMOs analysis, the energies of the HOMO and LUMO are valuable for calculating reactivity descriptors such as ionization potential (*I*), electron affinity (*A*), chemical hardness (*η*), global softness (*ω*), and chemical potential (*μ*), as defined by Koopman's theorem.^24,25^ The electron affinity (*A*) and ionization potential (*I*) can be calculated using eqn (2) and (3) (Table S3†).^26^ Meanwhile, global hardness (*η*), electronegativity (*X*), and chemical potential (*μ*) were calculated using eqn (4)–(6) (Table S3†).^27^ Electrophilicity (*ω*) was calculated to determine the charge transfer using eqn (7) (Table S3†), while global softness (*σ*) was calculated using eqn (8) (Table S3†).^28,29^

In Table 1, the computed values for ionization potential (*I*), electron affinity (*A*), global hardness (*η*), electronegativity (*X*), chemical potential (*μ*), electrophilicity (*ω*), and global softness are shown. The electron donor and acceptor behavior of materials can be described using the ionization potential (IP) and electron affinity (EA), respectively. The calculated values of IP and EA were 5.60 eV and 1.78 eV. The predicted value of electronegativity (3.69 eV) was significantly high. The stability and reactivity of the material can be predicted by global hardness (*η*), and the value of *η* for Cyperol A was 1.91 eV. Furthermore, the predicted values of chemical potential (*μ*), chemical softness (*σ*), and global electrophilicity index (*ω*) were −1.91, 0.52, and 0.95, respectively. All these molecular descriptors indicate that Cyperol A has greater biological potential compared to the BPH insecticide carbofuran.

**Table 1:** Calculated molecular descriptors of Cyperol A and carbofuran at the B3LYP/6-311G (d,p) level

Molecular descriptors	Cyperol A	Carbofuran
*E*_HOMO_ (eV)	−5.60	−8.35
*E*_LUMO_ (eV)	−1.78	−4.27
Δ*E*_HOMO–LUMO_ (eV)	3.82	4.08
Ionization potential, IP (eV)	5.60	8.35
Electron affinity, EA (eV)	1.78	4.27
Electronegativity, *χ* (eV)	3.69	6.31
Global hardness, *η* (eV)	1.91	2.04
Chemical potential, *μ* (eV)	−1.91	−2.04
Chemical softness, *σ* (eV^−1^)	0.52	0.49
Global electrophilicity index, *ω* (eV)	0.95	1.02
Dipole moment (debye)	5.41	6.09
Polarizability *α* (a.u)	125.05	137.78

### 2.2. Vibrational analysis

The theoretical FTIR bands and relative intensities of Cyperol A were computed at the DFT/B3LYP/6-311G (d,p) level to predict the vibrational modes (Table S2†).^30,31^

#### 2.2.1. C–H and O–H vibrations

In the experimental FTIR spectra, aromatic C–H stretching is observed between 3000–3100 cm^−1^ (ref. 32) while cyclic non-aromatic C–H symmetric stretching is observed between 2956 and 2852 cm^−1^.^33^ The computed O14–H15 stretching is found at 3814 cm^−1^, and C–H stretching vibrations for the aromatic region were found in the range of 3207 to 3208 cm^−1^, with stretching vibration for the aromatic C3–H1 and C5–H8 at 3208 and 3207 cm^−1^, respectively. The saturated five-membered fused heterocyclic carbon showed the H9–C6–H10 stretching vibrations at 3056 and 3115 cm^−1^. Furthermore, the aldehyde C11–H13 showed a stretching vibration at 2928 cm^−1^ for Cyperol A. The bending vibrations were found at 991, 1030, 1045, 1096, 1136, 1164, 1200, 1204, 1230, 1254, 1322, 1432, 1443, 1460, 1500, 1509, 1554 and 1641 cm^−1^ for Cyperol A.

#### 2.2.2. C=C stretching vibration

The experimental FTIR stretching vibration of the C=C bond is generally located between 1660 and 1600 cm^−1^ in FTIR spectra, while for the aromatic system, it is found between 1600 and 1475 cm^−1^.^34^ The stretching C=C vibration for the aromatic region was observed at 1693–1460 and 1322–1230 cm^−1^ for 1. The bending C=C vibrations were found at 395, 482, 494, 513, 608, 618, 702, 735, 760, 818, 832, 953 and 395 cm^−1^ for compound 1. The rotational vibrations were found at 1131, 1021, 833, 688, 612, 505, 408, 338, 245, 210, 196, 164, 158, and 101 cm^−1^ for Cyperol A.

#### 2.2.3. CHO vibration

The C=O stretching vibration is typically found between 1850 and 1600 cm^−1^.^35^ For Cyperol A, the C=O stretching vibration of the aldehyde group was located at 1768 cm^−1^. The C–H bending vibrations of the aldehyde group were located at 1443, 1432, 1021, 505, 408, and 196 cm^−1^. The rotational vibrations were found at 93, 152, 156, 199, 267, 290, 313, 354 and 395 cm^−1^.

#### 2.2.4. Vibrational comparison of Cyperol-A with insecticide carbofuran

**Fig. 5:**
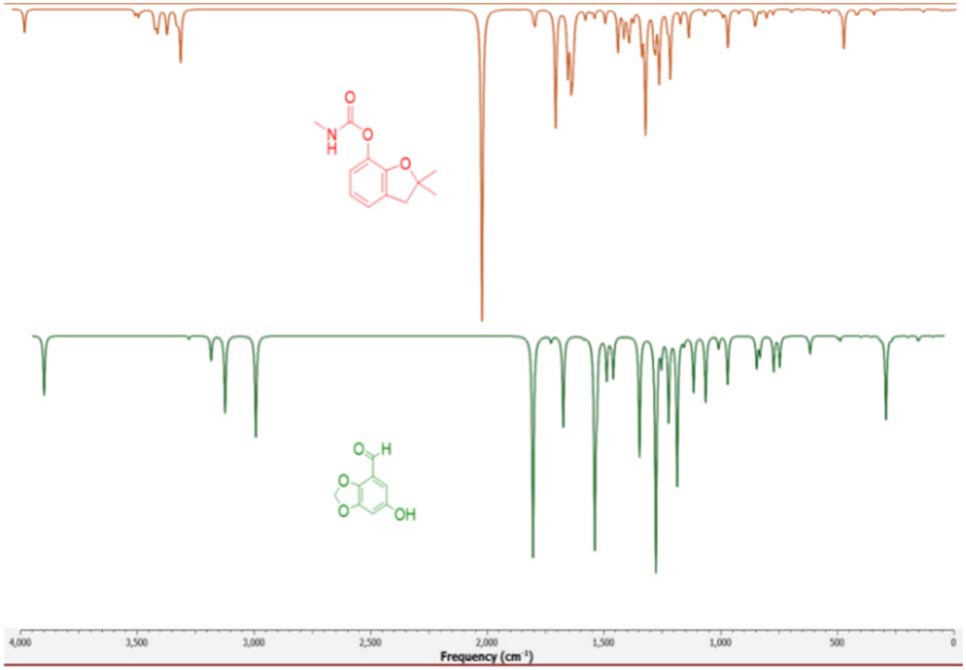
Comparison of the vibrational modes of Cyperol A with those of the insecticide carbofuran.

The excitation of molecules at the atomic level plays a key role in their activities and interactions with the surrounding systems. Therefore, we compare the vibrational modes of Cyperol A with those of carbofuran, specifically targeting its efficacy against brown planthopper using IR spectroscopy calculations. Similar vibrational patterns observed between Cyperol A and carbofuran suggest that Cyperol A could be a potential substitute for carbofuran. The vibrational modes of the methylene group (3086–3056 cm^−1^) and the benzene methine group (3200–3100 cm^−1^), as well as the NH (3647 cm^−1^) and OH (3814 cm^−1^) stretches, show similar patterns (Fig. 5).

### 2.3. Time- and dose-dependent toxicity of compound 1

Previously, for carbofuran, the highest mortality of 73.3% was recorded at a dosage of 0.5 kg ai ha^−1^, which was effective in controlling the brown planthopper (BPH) at 72 hours after treatment (HAT).^36^ Similarly, carbofuran exhibited significant dose- and time-dependent mortality against the brown planthopper (*Nilaparvata lugens*), with 90.0% mortality observed 24 hours after treatment (HAT), increasing to 100% at 48 HAT and remaining consistent at 72 HAT.^37^ The primary challenge in controlling the brown planthopper (*Nilaparvata lugens*) is the development of insecticide resistance. Different studies have reported varying LC_50_ values for insecticides against *N. lugens*. In one study, the LD_50_ value for carbofuran was reported to be 20.3 μg g^−1^, with a 95% confidence interval ranging from 13.4 to 91.1 μg g^−1^.^38^

A diverse group of isolated natural compounds, present in numerous plant species, exhibits a wide range of biological activities. Many natural compounds have gained attention as promising botanical insecticides due to their potent antifeedant and insecticidal properties against various insect pests.^39^ Only a few rare natural compounds isolated from plants have been demonstrated to possess anti-complement and antifeedant activities.^40^ However, this study represents the first report on the contact toxicity of naturally isolated Cyperol A against *N. lugens*.

**Fig. 6:**
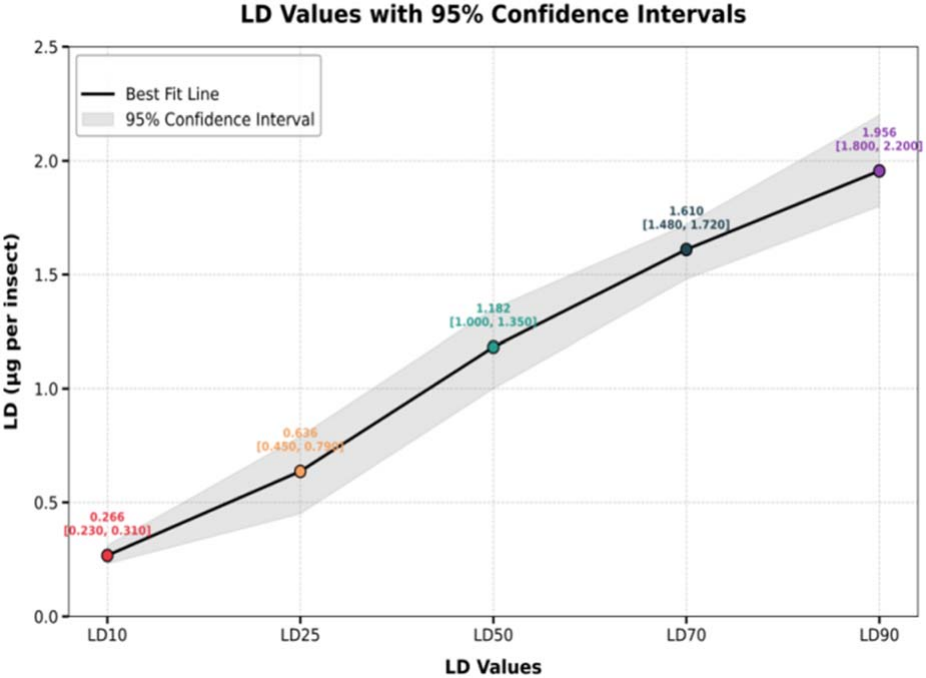
LD_10_, LD_25_, LD_50_, LD_70_ and LD_90_ values with 95% confidence interval.

In the current study, compound 1 exhibited potent insecticidal activity, characterized by both time- and dose-dependent toxicity, making it a highly effective candidate for pest control. The LD_50_ value of 1.18 μg per insect (95% CI: 0.89–1.38) underlines its potency, achieving 50% mortality at relatively low doses. Even minimal concentrations are toxic, as evidenced by the LC_10_ and LC_25_ values of 0.26 μg per insect and 0.63 μg per insect, respectively. At higher doses, the efficacy is enhanced, with LC_70_ and LC_90_ values of 1.61 μg per insect and 1.95 μg per insect, respectively. The steep slope of the Hill (1.158) signifies a sharp increase in mortality with increasing doses, which is characteristic of potent insecticides. Moreover, the high *R*-squared value of 0.9368 validates the reliability of the data, confirming the compound's robust dose–response relationship. These findings highlight the efficiency of compound 1 in achieving significant insect mortality, providing flexibility in dose application based on pest severity (Fig. 6).

**Fig. 7:**
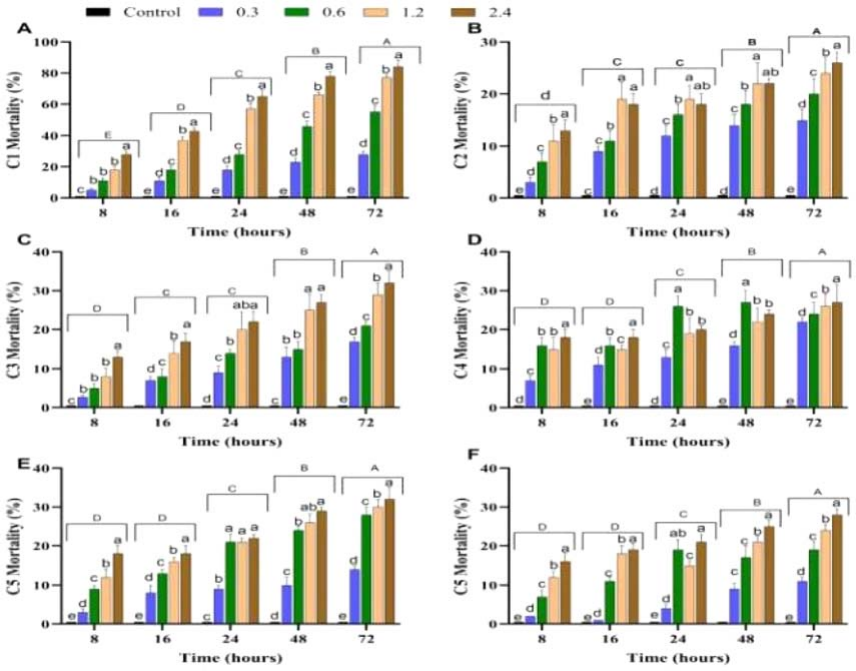
Time- and dose-dependent mortality percentages of compounds 1–6 (A–F). Different lowercase and uppercase letters above the bars indicate statistically significant differences (*p* < 0.05) among various doses and time intervals, respectively, as determined by Tukey's test. The *X*-axis represents time intervals (8, 16, 24, 48, and 72 hours). Error bars represent the mean ± standard deviations (SD).

There is also a marked time dependency, with mortality rates progressively increasing over time. At lower time points, such as 8 hours, mortality is modest, even at higher doses. However, with prolonged exposure at 24, 48, and 72 hours, the mortality rates rise sharply, demonstrating the prolonged efficacy of the compound. For instance, at 1 μg per insect, mortality increases from approximately 20% at 8 hours to almost 70% at 72 hours (Fig. 7).

This comparison highlights the superior efficacy of compound 1 as an insecticide against *N. lugens* and underlines its potential for effective pest control applications in agriculture.

In contrast, compounds 2 to 6 exhibited significantly lower mean mortality percentages across all dose levels. When the LD_50_ value exceeds 2 μg per insect, it is categorized as non-toxic.^41^ In our study, compounds 2–6 are classified as non-toxic based on their mortality results, as their LD_50_ values exceed 2 μg per insect.

For instance, at the highest tested dose (2.4 μg per insect), compound 2 achieved only 16% mean mortality, while compound 6 achieved 21.8%. This comparison highlights the superior efficacy of compound 1 as an insecticide against *N. lugens* and underlines its potential for effective pest control applications in agriculture.

### 2.4. Time- and dose-dependent AChE inhibition by compound 1

The brown planthopper (*Nilaparvata lugens*) exhibits significant insecticide resistance due to reduced AChE sensitivity. The IC_50_ values previously reported for carbofuran are 1.5 × 10^−2^ mM and 8.4 × 10^−2^ mM in the resistant Rc-30 and Rf-30 strains, respectively, compared to 7.8 × 10^−4^ mM in the susceptible (*S*) strain. These values correspond to 19.2- and 107.7-fold insensitivity ratios, indicating a marked reduction in carbofuran efficacy against resistant populations.^38^

In our study, compound 1 demonstrated the most potent acetylcholinesterase (AChE) inhibition, exhibiting strong dose- and time-dependent effects compared to other compounds. At the LD_50_ dose (1.182 μg per insect), the inhibition rate increases progressively over time, starting at 19.3% at 0.5 hours and peaking at 79% at 24 hours. This trend highlights the prolonged effect and significant inhibitory potential of compound 1, indicating that it maintains high efficacy over extended periods. Its superior performance at all time intervals reflects its ability to efficiently disrupt AChE activity, making it highly effective as a pesticide.

In contrast, compounds 2–6 exhibit considerably weaker AChE inhibition. For instance, at 0.5 hours, compound 2 inhibits AChE by only 9%, while compound 6 achieves only 7%. At 24 hours, the inhibition rates for these compounds remain significantly lower, with compound 2 reaching 26% and compound 6 achieving 25%, both substantially below 79% of compound 1. Similarly, compounds 3–5 display lower inhibition rates and do not match the effectiveness of compound 1 across all time intervals. This comparative analysis clearly identifies compound 1 as the most promising candidate for pest control due to its robust and sustained AChE inhibition over time, which is critical for the effective disruption of the insect nervous system function.

### 2.5. Time and dose-dependent inhibition of glutathione S-transferase (GST)

Detoxification enzymes, including carboxylesterases (CarE), acid and alkaline phosphatases (ACP and ALP), cytochrome P450 monooxygenases (P450s), and glutathione S-transferases (GSTs), are crucial for neutralizing toxic compounds.^10^ A reduction in the activity of these enzymes is closely linked to a decreased ability of insects to metabolize and eliminate insecticides.^42^ Previous research indicates that exposure to certain compounds, such as kaempferol-3-*O*-glu-rha-glu, quercetin-3-*O*-glu-rha-glu,^43^ and pectolinarigenin,^44^ can significantly inhibit the activities of P450s and GSTs, which are phase I detoxification enzymes. Such inhibition weakens the detoxification capacity and reduces the metabolic ability of *N. lugens*. These effects correlate with the increased mortality in insects exposed to insecticides.^45^

**Fig. 8:**
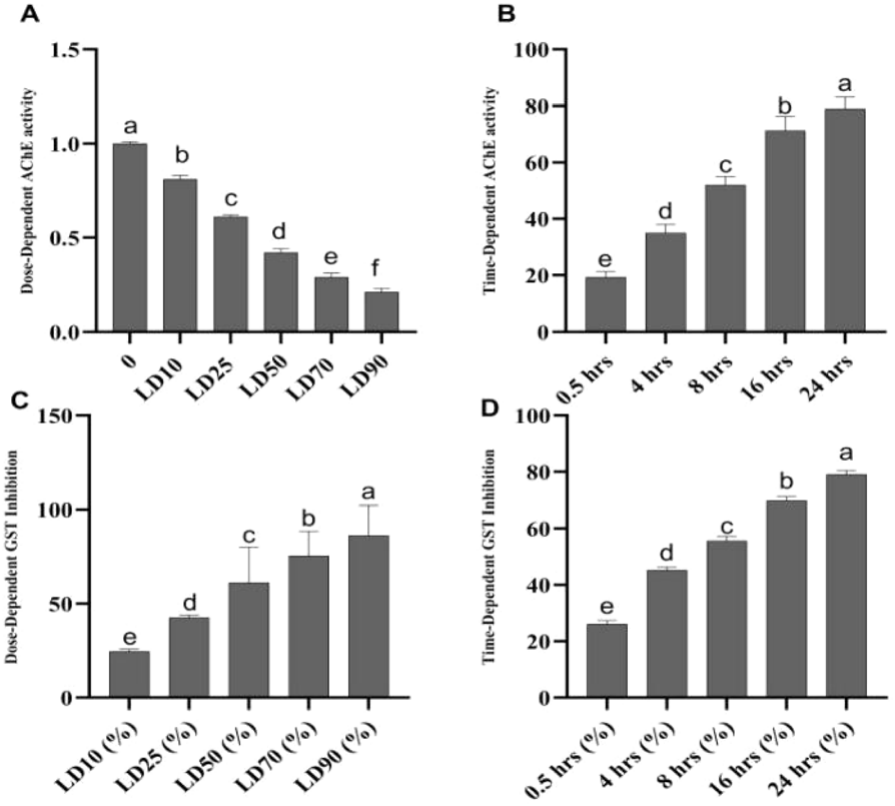
(A) Dose-dependent acetylcholinesterase (AChE) inhibition by compound 1 (C1), (B) time-dependent AChE inhibition by C1, (C) dose-dependent glutathione S-transferase (GST) inhibition by C1, and (D) time-dependent GST inhibition by C1. Different letters above the bars indicate statistically significant differences (*p* < 0.05) as determined by Tukey's test. Error bars represent the mean ± standard deviations (SD).

In the dose-dependent assay, compound 1 exhibited the strongest inhibition at all doses tested, with inhibition reaching 86.3% at LD_90_, demonstrating its high potency and efficacy. The inhibition was consistent across doses, and its variability was low (±1.6% SD). Compound 2 showed moderate inhibition, with a peak of 48.5% at LD_90_, while compound 3 displayed the weakest inhibition, reaching only 36.9% at the same dose. Compounds 4–6 showed intermediate inhibition, with values ranging from 43.2% (compound 4) to 41.7% (compound 6) at LD_90_ (Fig. 8).

In the time-dependent assay, compound 1 again exhibited the strongest inhibition, achieving 79.1% at 24 hours with low variability (±1.3% SD). The other compounds showed a gradual increase in inhibition over time. Compound 2 reached 43.2% at 24 hours, while compounds 3–6 exhibited relatively weaker inhibition, with 28.1% (compound 3) to 38.8% (compound 4) at the same time point.

The exceptional performance of compound 1 in both assays highlights its potential as a highly effective insecticide. Its potent and consistent inhibition of GST makes it a promising candidate for insecticide development, offering rapid and sustained activity. The potent insecticidal effects of compound 1 could be pivotal in developing more efficient and eco-friendly pest management strategies, potentially reducing reliance on traditional chemical insecticides and contributing to sustainable agricultural practices. Further research into optimizing the formulation and application of compound 1 could enhance its utility in integrated pest management (IPM) systems, ensuring long-term control of pests like the brown plant hopper (BPH).

## 3. *In silico* studies

**Fig. 9:**
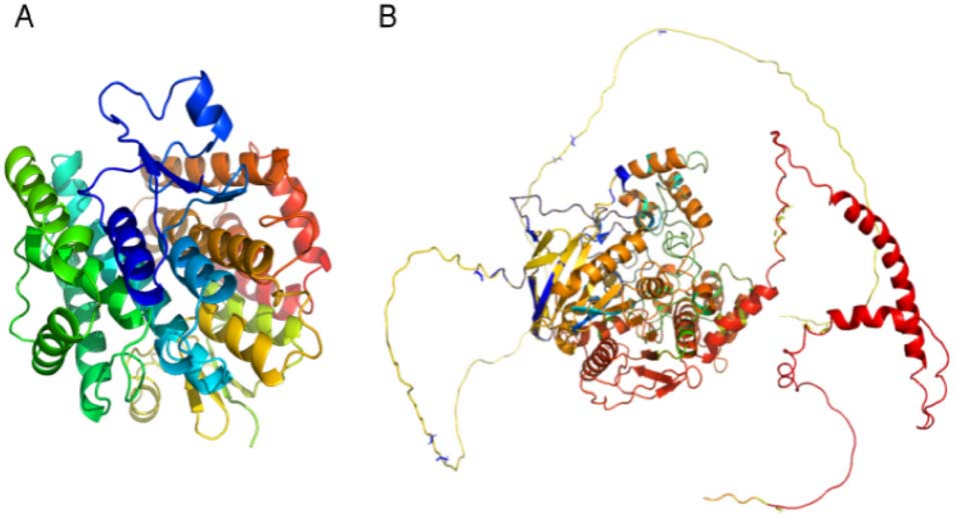
Three-dimensional (3D) structures of *N. lugens* enzymes: (A) glutathione S-transferase (GST) from the protein data bank (PDB), (B) acetylcholinesterase (AChE) from AlphaFold.

Plant-derived substances are eco-friendly and effective against insects, making them potential alternatives to synthetic insecticides. Finding new substances that bind molecular targets could be made easier and faster through molecular docking. Insects have important detoxifying enzymes such as glutathione S-transferase (GST), acetylcholinesterase (AChE), and cytochrome P450. This enzyme system (CYP450) contributes to their resistance to several insecticides and environmental stresses; thus, targeting these enzymes is a promising approach for the development of new insecticides (Fig. 9).^46,47^

The glutathione S-transferase of *N. lugens* structures was retrieved from the Protein Data Bank (PDB) database (PDB code: 3WYW) and Acetylcholinesterases (AChE) with accession number AF-G9BJC1-F1. The structural model was obtained from the AlphaFold database (https://www.AlphaFold.org).^46^ Polar hydrogen atoms were added to the protein and ligand by MOE (Molecular Operating Environment version 2019.0102), and energy minimization was applied to prepare them for docking, followed by 3D protonation.^47^

### 3.1. Validation of the modelled structures

Different types of stereochemical parameters of the protein structure were predicted online using PROCHECK, ERRAT, and Verify3D (https://www.saves.mbi.ucla.edu). PROCHECK analyzes the overall model geometry by generating the Ramachandran plot with favorable and non-favorable residue regions. ERRAT is a database of highly refined protein structures that uses a nine-residue sliding window to assess the residue *versus* error relationship. Verify3D validates a 3D structure in terms of loops, sheets, and alpha helices.^48,49^

**Fig. 10:**
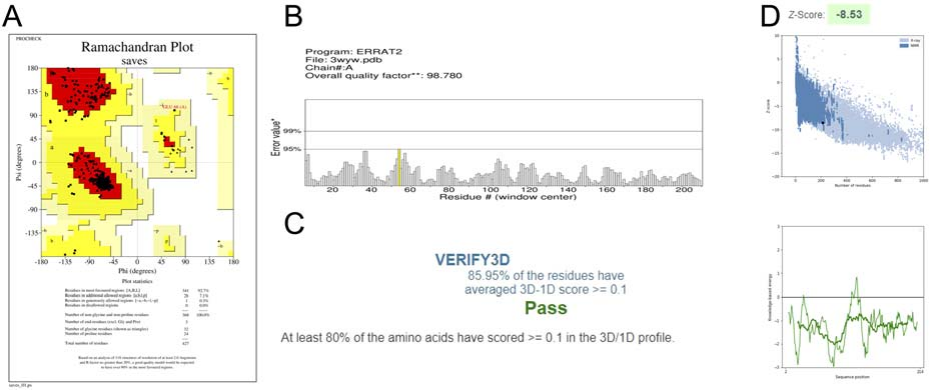
Validation of the 3D structure of *N. lugens* GST using: (A) Ramachandran plot from PROCHECK, indicating the most favorable, favorable, and disallowed regions; (B) the ERRAT server quality factor, showing error rates below the 95% rejection threshold; (C) Verify3D analysis; and (D) ProSA-web *Z*-score.

**Fig. 11:**
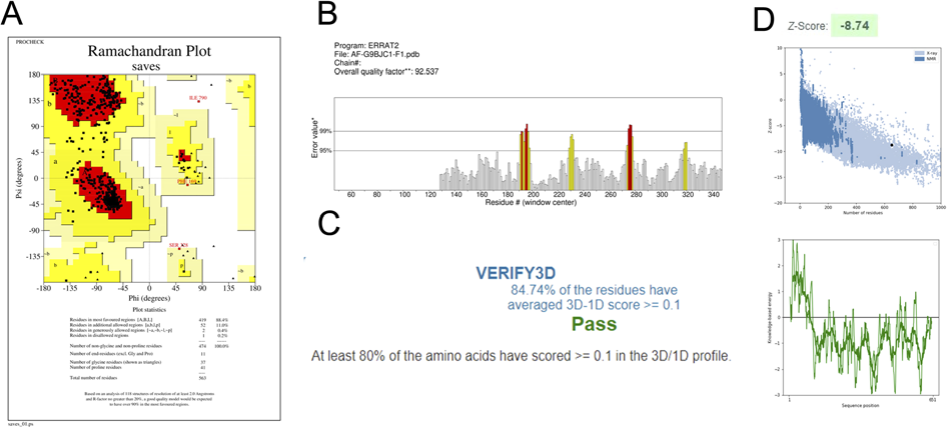
Validation of the 3D structure of *N. lugens* acetylcholinesterase (AChE) using: (A) Ramachandran plot from PROCHECK, indicating the most favorable, favorable, and disallowed regions; (B) ERRAT server quality factor, showing error rates below the 95% rejection threshold; (C) Verify3D analysis; and (D) ProSA-web *Z*-score.

The Ramachandran plots for GST and AChE are given in Fig. 10A and 11A. In ERRAT, the GST and AChE structures show overall quality factors of 98.78 and 92.53, respectively (Fig. 10B and 11B). In Verify3D model verification, where the minimum passing score was 80%, the 3D models received scores of 85.95 and 84.74% for GST and AChE (Fig. 10C and 11C). The ProSA-web server was utilized to compare the protein structural models to the PDB's protein structures based on *Z*-score.^50^

A plot of the residual energy and the input structure's *Z*-score was provided by the application SWISS-MODEL. The *Z*-score showed *Z*-scores of −8.53 and −8.74 for GST and AChE (Fig. 10D and 11D), which represent excellent models.

### 3.2. Active site prediction

Docking efficiency is significantly increased when the location of the active sites or binding sites is known before the docking procedures. Cavity detection tools can be used to find probable active sites within proteins. Blind docking is the process of docking without making any assumptions about the binding location.^51^ The type of interactions between protein molecules dictates their biological roles, which involve a few residues and binding sites where the interaction occurs. One of the most important steps in finding new chemical entities in molecular docking for structure-based drug design is identifying the binding site, which is accomplished by examining the physicochemical and shape properties of the protein area. The target proteins and binding sites with little to no ability to bind ligands can be excluded from the binding site identification process.^52,53^

Twenty-six high-potential binding sites were found in GST and the one that has the following amino acids was chosen: VAL8, PRO9, GLY10, SER11, ALA12, PRO13, THR33, ASP34, LEU35, LYS36, HIS40, GLN51, HIS52, ASN53, VAL54, PRO55, ASN65, GLU66, SER67, ARG68, MET103, TYR107, GLN108, GLY111, ASP112, TYR115, PRO116, PHE119, LYS128, GLU201, GLY202, GLY205, PHE206, GLN208, MET209, TYR100, GLY104, THR105 GLN108, SER109, ASP112, PRO116, ASP127, LYS128, LYS131, ASP134, ALA135 and PHE138.

Furthermore, 30 binding sites in AChE were analysed, and focusing on the binding sites with amino acids ILE199, ASP201, SER210, TRP213, PHE245, GLY246, GLY247, GLY248, TYR250, SER251, GLY252, THR253, LEU256, TYR259, ALA280, GLU327, SER328, ALA329, GLY330, ALA331, TRP409, GLY410, THR411, LEU412, GLY413, ILE414, CYS415, GLU416, PHE417, TYR457, PHE458, TYR461, HIS462, HIS568, GLY569 and ILE572 were selected. Previously, azadirachtin was found to interact with acetylcholinesterase of *Tribolium castaneum*.^54,55^

**Fig. 12:**
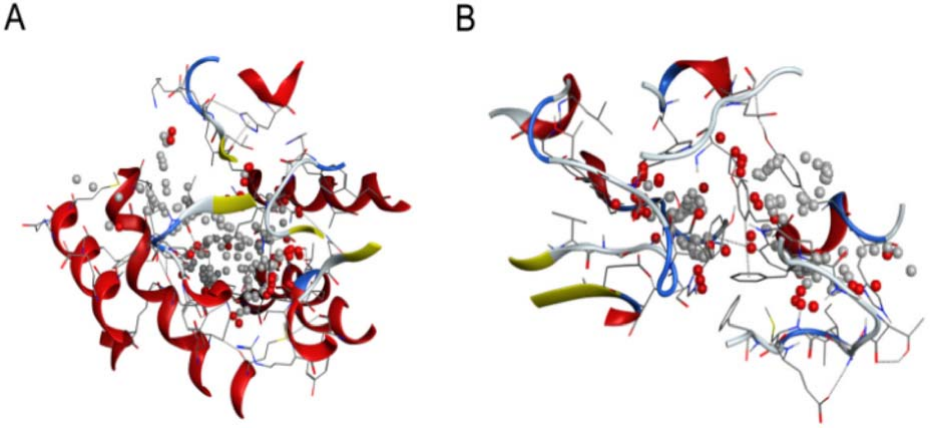
(A) Three-dimensional structure of glutathione S-transferase (GST) and (B) three-dimensional structure of acetylcholinesterase (AChE), where the active sites are shown by tiny red and grey dots.

Moreover, an activity prediction test was also employed, following the method described by Empereur-Mot *et al.*, to confirm the modelled protein's active site.^56^ Finally, the new isolated compound was accurately docked into the proposed active sites, as shown in Fig. 12 (Table 2).

**Table 2:** Top five docking poses of Cyperol A with GST from *N. lugens*

S. no.	mol	rseq	mseq	*S*	rmsd
1	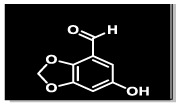	1	1	−9.7581	1.9837
2	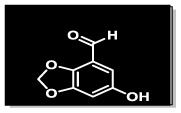	1	1	−9.4288	0.7362
3	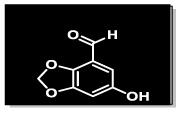	1	1	−9.4016	2.1250
4	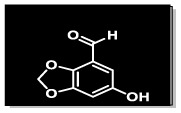	1	1	−9.0724	1.6885
5	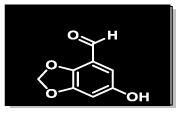	1	1	−8.9804	1.6166

### 3.3. ADME (absorption, distribution, metabolism, and excretion) analysis

The Swiss ADMET online tool was utilized to predict the absorption, distribution, metabolism, elimination and pharmacokinetic properties of molecules to evaluate the Lipinski five rules (MW, iLOGP, HBAs, and HBDs).^57^ MW was recorded as 166.13 g mol^−1^, and log *P* was 1.33. Cyperol A presented no violations, and according to Lipinski's rule of five, it is the best drug candidate.

### 3.4. Molecular docking

Molecular docking stimulates interactions between the ligand and the protein at the atomic level, clarifies basic biochemical processes, and characterizes the behavior of small molecules at the target protein binding sites. The two fundamental steps in the docking process are predicting the ligand's orientation and position within these sites, also known as pose, and evaluating the binding affinity.^58^ The most crucial element of the structure-based drug design is the scoring function. The scoring functions are mathematical functions used to roughly forecast their binding affinities. In the ligand–receptor interactions, the lowest scores are ideal and are frequently utilized in virtual screening and drug discovery.^59^ The outcomes of our docking study demonstrated that the chosen inhibitor docked within the pockets of *N. lugens* AChE and GST target proteins has potential interactions with AChE and GST. Following the *in silico* docking, the lowest *S* score of Cyperol A with GST was assessed, which established the strongest interaction with a minimum *S* score of −9.75, followed by −9.42 (Table 2). AChE shows strong and the best interactions with Cyperol A, with a docking score of −10.83, followed by −9.97, as shown in (Table 3). This indicates the best docking score and shows the capability of Cyperol A in insecticide development.

**Table 3:** Top five docking poses of Cyperol A with AChE from *N. lugens*

S. no.	mol	rseq	mseq	*S*	rmsd
1	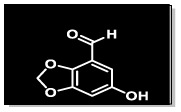	1	1	**−10.8313**	**0.8641**
2	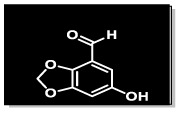	1	1	−9.9793	2.5001
3	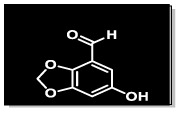	1	1	−9.2352	1.3641
4	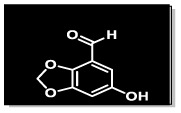	1	1	−9.1782	0.6822
5	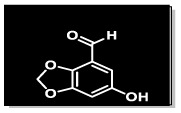	1	1	−8.5863	1.8961

### 3.5. Protein–ligand interaction

**Fig. 13:**
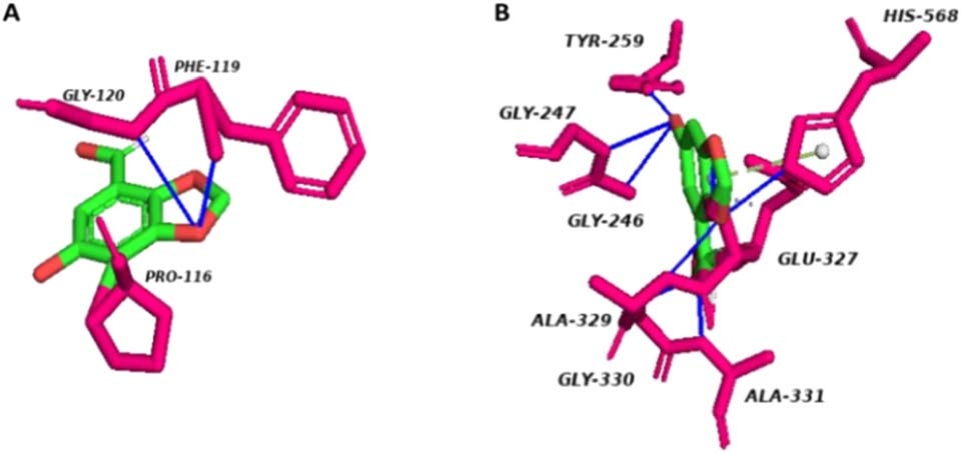
(A) Cyperol A interactions with GST. (B) Cyperol A interaction with AChE.

PLIP (https://plip-tool.biotec.tu-dresden.de) was used to map the interaction of GST and AChE with Cyperol A. Cyperol A formed hydrogen bonds with two specific amino acids (PHE119 and GLY120) in its interaction with GST. The bond distances were measured to be 2.39 and 3.21 Å. Cyperol A also formed one hydrophobic interaction with the amino acid PRO116 with GST (Fig. 13A).

Nine hydrogen bonds and two hydrophobic interactions were also found between Cyperol A and AChE. The observed hydrogen bonds with amino acids GLY246A, GLY247, TYR259, TYR259, SER328, ALA329, GLY330, ALA331, and HIS568, with bond distances measuring 3.07, 4.0, 2.61, 2.61, 2.18, 2.93, 2.37, 2.36 and 1.91 Å, respectively. Hydrophobic interactions of Cyperol A were also found with amino acids GLU327 and SER328 (Fig. 13B).

## 4. Method and materials

### 4.1. General experimental

The JASCO A-302 and Shimadzu UV-240 spectrophotometers were used to record the IR and UV spectra, respectively, while the JASCO P-2000 Polarimeter was used to record the specific rotations of all the isolated compounds. ^1^H and ^13^C-NMR spectra were taken on Bruker Avance NMR spectrometers (300, 400, and 500 MHz). Finnigan MAT 95 XP, Finnigan MAT 311, and Jeol JMS HX 110 mass spectrometers were used for low-resolution EI, HREI-MS, HRMS Q-Exactive MS-US Thermo Fisher Scientific, and FABMS investigations, respectively. Silica gel (70–230 mesh, Kieselgel 60) was used for the column chromatography, and TLCs were performed on F_254_ aluminium sheets (0.25 mm).

### 4.2. Plant material

The *C. rotundus* plant material was collected from the Okara region, Punjab, Pakistan, and a voucher specimen (A.R., no. 112) was submitted to the Department of Botany's Herbarium of Government College University Faisalabad, Pakistan.

### 4.3. Extraction and isolation

The plant was dried under the shade and powdered. The dried powdered material was then soaked in 95% methanol at room temperature (3 × 5 days × 25 L) to obtain 300 g of crude extracts of *Cyperus rotundus*.

#### 4.3.1. Purification from *Cyperus rotundus*

##### 4.3.1.1 Vacuum liquid chromatography

The crude extract of *C. rotundus*, weighing 300 g, was subjected to vacuum liquid chromatography using an ethyl acetate/*n*-hexane solvent system and 1.0 kg of silica gel to obtain fractions (I–V): (Fr. I, 0 : 10), (Fr. II, 1 : 9), (Fr. III, 2 : 8), (Fr. IV, 4 : 6), and (Fr. V, 6 : 4).

#### 4.3.2. Repeated column chromatography-I

Normal phase column chromatography was performed for Fr. V (24.7 g), using a normal phase silica gel column (700 g, 4 × 60 cm) to obtain four major sub-fractions with acetone/*n*-hexane: (Fr. V1, 1 : 9), (Fr. V2, 2 : 8), (Fr. V3, 3 : 7), and (Fr. V4, 5 : 5).

Fraction V3 (5.3 g) was further subjected to column chromatography to get three fractions using pure methanol as the solvent system in a Sephadex (LH-20) column (500 g, 3 × 60 cm): (Fr. V3A, 1 : 9), (Fr. V3B, 3 : 7), and (Fr. V3C, 2 : 3). A new compound 1 (178 mg) was purified from Fr. V3A (965 mg) through normal phase column chromatography in an acetone/*n*-hexane 3 : 7 solvent system using 20 g of normal phase silica gel in a column (1 cm × 10 cm). Compound 2 (57 mg) and compound 3 (20.5 mg) were purified from Fr. V3C (744 mg) by normal phase column chromatography using 100 g silica gel in a column (1 cm × 40 cm) with an acetone/*n*-hexane 5 : 5 solvent system.

#### 4.3.3. Repeated column chromatography-II

The major Fr. II (37 g) was subjected to column chromatography with an ethyl acetate/*n*-hexane solvent system in a column (8 cm × 60 cm) using 800 g of normal phase silica gel to get five fractions: (IIA, 0 : 4), (IIB, 1 : 4), (IIC, 2.5 : 3.5), (IID, 2.5 : 2.5), and (IIE, 3.5 : 1.5). Fr. IIB (7.4 g) was fractionated using 300 g of silica gel in a column (3 cm × 40 cm) to get three minor fractions: (IIB1, 0 : 4), (IIB2, 1 : 4), (IIB3, 1.5 : 3.5), and (Fr. IIB4, 2 : 3). Fr. IIB3 (1.6 g) was fractionated into five minor fractions using 200 g of silica gel with column: (2 cm × 40 cm) (IIB3A, 0 : 4), (IIB3B, 1 : 4), (IIB3C, 1.5 : 3.5), (IIB3D, 2 : 3), and (IIB3E, 2.5 : 2.5).

#### 4.3.4. Normal phase HPLC (ethyl acetate/*n*-hexane)

Compound 4 (49 mg), compound 5 (30.1 mg) and compound 6 (19.2 mg) were purified from the Fr. IIB3E (99 mg) by following the normal phase HPLC (LH-80) in an ethyl acetate/*n*-hexane 9 : 1 solvent system (Fig. 1).

##### 4.3.4.1 Cyperol A (1)

Yellowish amorphous solid; m.p.: 186–188 °C, IR (CHCl_3_) *λ*_max_ cm^−1^: 3400.1 cm^−1^ (OH), 1733 cm^−1^ (C=O); UV *λ*_max_ nm (MeOH) (log *ε*): 305 (2.8); EI-MS: *m*/*z* [M]^+^ = 166.13 (calculated 166.13 for C_8_H_6_O_4_). The calculated HRMS of the compound, C_8_H_6_O_4_, is 166.02606 while the negative ion mode, high-resolution mass spectrometry (HR-ESIMS) performed on a Q-Exactive Mass Spectrometer (Thermo Fisher Scientific, USA) shows a prominent peak at 165.01755.

^1^H NMR (400 MHz, CDCl_3_): *δ*_H_ 6.20 (2H, s, H-2), 7.24 (1H, s, H-5), 7.33 (1H, s, H-7), *δ*_CH̲O_ 10.21 (CH̲O, s, H-8), *δ*_OH̲_ 7.52 (OH̲-6, s, HO-8) and ^13^C-NMR (125 MHz, CDCl_3_): *δ*_C_ 151.6 (C-1a), 104.0 (C-2), 147.3 (C-3a), 128.3 (C-4), 107.7 (C-5), 152.3 (C-6), 105.3 (C-7), 186.9 (C-8).

### 4.4. DFT calculation

The quantum chemistry computations were performed using the Gaussian 16W software suite on a Dell PC with a 12th Gen Intel® Core™ i7-1255U and 24 GB of RAM.^60^ The molecular geometry of compound 1 was fully optimized. The B3LYP/6-311G (d,p) method was used to compute the theoretical vibrational spectra. The NBO 3.1 program,^61^ at the B3LYP/6-311G (d,p) level, was used to construct the natural bond orbital (NBO) and to calculate the intermolecular delocalization or hyperconjugation through second-order interactions between the occupied and unoccupied orbitals of the system. The TD-DFT approach was employed to calculate the electronic characteristics, such as the highest occupied molecular orbital (HOMO) and the lowest unoccupied molecular orbital (LUMO).

## 5. Insect mortality and enzyme inhibition assay

*N. lugens* was originally obtained from the Integrated Pest Management Laboratory, Department of Entomology, University of Agriculture, Faisalabad, Pakistan. The *N. lugens* was reared continuously on rice seedlings at 25 ± 1 °C and 70% ± 5% relative humidity with a photoperiod of 16 : 8 h (light : dark). Insecticidal activities of the six isolated compounds from *C. rotundus* against *N. lugens* were determined by a micro-topical toxicity bioassay using a hand microapplicator (Burkard Manufacturing Co., Rickmansworth, England).^62,63^ Each test material was diluted into a series of concentrations with acetone and applied to 3 to 5 day-old adult insects. Thirty insects were lightly anaesthetized with carbon dioxide. Subsequently, a droplet (0.30, 0.60, 1.20, and 2.40 μg per insect) of each sample solution was applied topically with a hand microapplicator to the mid-abdomen of each insect. Control planthoppers (*N. lugens* adults) were treated with acetone only. The treated *N. lugens* were maintained with rice seedlings in a glass tube at 25 ± 1 °C, 65–75% relative humidity, and a light : dark photoperiod of 16 : 8 h. The mortalities were recorded after treatment at 8, 16, 24, 48, and 72 h, and all treatments were replicated three times.

### 5.1. Enzyme extraction

The insects were homogenized on ice in a cold homogenization buffer specific for AChE activity. For the AChE assay, a 0.1 M sodium phosphate buffer (pH 7.6) was used. After homogenization, the samples were centrifuged at 3500 rpm for 10 minutes at 4 °C to obtain the supernatant using a TGL-16 M freezing centrifuge (Hunan Xiangyi, China). The clear supernatants were collected and stored at −80 °C. These supernatants were used as crude enzyme extracts for AChE activity analysis.

### 5.2. AChE activity assay

The AChE activity was determined using acetylthiocholine iodide (ATCI) as the substrate.^64^ In a 96-well microplate, 50 μL of crude enzyme extract was mixed with 100 μL of 0.1 M sodium phosphate buffer (pH 7.6) and 50 μL of ATCI solution (1 mM final concentration). The reaction was initiated by adding the substrate, and the plate was incubated at 25 °C for 10–15 minutes.

### 5.3. Measurement

The reaction was stopped by adding DTNB (5,5′-dithiobis(2-nitrobenzoic acid)) to a final concentration of 0.5 mM. The DTNB reacts with the thiocholine produced by AChE activity to form a yellow product. The absorbance was measured at 412 nm using a Shimadzu UV-2650 spectrophotometer. The six test compounds were dissolved in acetone (final concentration ≤1%) and tested at varying concentrations. The inhibitory effects of these compounds on AChE activity were evaluated by comparing the absorbance in the presence of the test compounds with the control (acetone).

The percentage of enzyme inhibition was calculated using the formula:
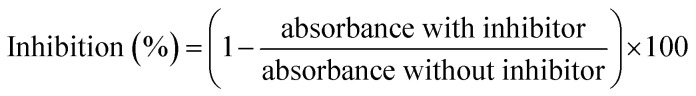


### 5.4. Measurement of cellular glutathione (GSH/GSSG) levels

The cellular glutathione content was quantified using a commercial assay kit obtained from Nanjing Jiancheng Bioengineering Institute, China. Insects were homogenized to a 20% (w/v) concentration on ice in the provided reagent IV from the kit. The homogenates were then subjected to centrifugation at 3500 rpm for 10 minutes at 4 °C to obtain the supernatant, which was clear and used for subsequent analysis. The glutathione concentrations (GSH and GSSG) were determined by measuring the absorbance, which was then converted to micromolar (μM) values using a standard curve prepared with known glutathione concentrations. Each experimental treatment was performed with three replicates to ensure statistical reliability.

### 5.5. Statistical analysis

All absorbance measurements were performed in triplicate for each treatment. The data were analyzed using GraphPad Prism to generate dose–response curves and calculate IC_50_ values. A two-factor ANOVA was performed to evaluate the time- and dose-dependent mortality percentages for compounds 1–6, followed by Tukey's test for post-hoc comparisons, with a significance level set at *p* < 0.05. Figures were prepared using GraphPad Prism (Premium Version 10). Enzyme activity values for each compound at different time points and concentrations were expressed as mean ± standard deviations (SD).

## 6. *In silico* study

### 6.1. Validation of the modeled structures

To optimize the 3D model structures, PROCHECK was used to create a Ramachandran plot analysing the phi and psi distribution of non-glycine and non-proline residues. The ERRAT services provided a quality factor, and Verify3D was also used to check the effectiveness of the structure. A plot of the residual energy and the input structure's *Z*-score using the ProSAweb server was provided by the application SWISS-MODEL's *Z*-score to compare the protein structures with the PDB.

### 6.2. Prediction of the active site

In molecular docking studies, identifying binding sites in the modelled protein structures is crucial for predicting potential receptor binding. The MOE site finder tool was employed to discover viable binding sites within the chosen proteins.

### 6.3. Lipinski's rule of five for drug-likeness or ADME (absorption, distribution, metabolism, and excretion) analysis

Lipinski's rule of five directs new drug development, ensuring optimal interaction with the target proteins or enzymes.^65^ Drug metabolism and pharmacokinetics (DMPK) research is pivotal but intricate, frequently resulting in elevated clinical trial failures.^66^ SwissADME was used to evaluate the absorption, distribution, metabolism, and excretion to assess drug suitability.^67^

### 6.4. Molecular docking

MOE (Version 2019.0102) was selected for molecular docking due to its intuitive graphical interface, which visualizes the binding residue locations for ligands and receptors. MOE organizes potential binding geometries based on the *S*-score, a numerical parameter offering a graphical data representation. The *S*-score is influenced by interactions with solvents, cations, and sulfur lone pairs.^68^ MOE is used to screen the binding of Cyperol A with GST and AChE. The generalized Born solvation model (GBVI) score function was applied in conjunction with the docking outcomes. A force field-based scoring method, GBVI/WSA dG, calculates the ligand's binding free energy from specific orientations. The *S* score was used to analyze the interaction outcomes.

## 7. Conclusion

The current research was aimed at the photochemical investigation of *C. rotundus* plant, resulting in the isolation of one new natural compound, together with five known compounds. The structure of the new compound was determined as Cyperol A by spectroscopic methods, including NMR (^1^H, ^13^C, COSY, HMBC, HSQC, NOESY) and EIMS. The newly isolated Cyperol A exhibits a stable structure, allowing further investigation. Additionally, the newly isolated Cyperol A was studied using DFT to determine its electronic features. The *in vitro* assessment of the insecticidal potential of compounds 1–6 against *Nilaparvata lugens* revealed that compound 1 exhibited remarkable toxicity and significant inhibition of key enzymatic activities. Moreover, the *in silico* study of a new natural compound with GST and AchE have also been carried out to determine the efficacy of Cyperol A as a biological pesticide targeting GST and AChE. The findings emphasize the intrinsic insecticidal properties of this new natural compound and its potential as an eco-friendly alternative to synthetic insecticides. Given the environmental concerns associated with conventional insecticides and the emergence of insecticide resistance in pests, natural compounds from plants like *C. rotundus* L. offer a sustainable and environmentally conscious approach to pest management. Additionally, exploring the molecular properties of these compounds may unveil novel applications in insecticide discovery and allied domains.

## Data availability

The data supporting this article have been included as part of the ESI.†

## Author contributions

Data curation, Saqib Hussain Bangash, Chen-Yang Wei and Wen-Wei Tang; formal analysis, Saqib Hussain Bangash, Akbar Ali and Wen-Wei Tang; investigation, Saqib Hussain Bangash, Amjad Hussain, Rashad Al-Salahi and Wen-Wei Tang; methodology, Saqib Hussain Bangash, Moazama Riaz and Wen-Wei Tang; resources, Saqib Hussain Bangash, Rashad Al-Salahi, Wen-Wei Tang; software, Saqib Hussain Bangash, Muhammad Fayyaz ur Rehman, Faiz Ahmed; supervision, Muhammad Ibrahim and Wen-Wei Tang; validation, Saqib Hussain Bangash, Akbar Ali; visualization, Saqib Hussain Bangash and Chen-Yang Wei; writing – original draft, Saqib Hussain Bangash and Wen-Wei Tang; writing – review & editing, Chen-Yang Wei, Rashad Al-Salahi, Faiz Ahmed, Akbar Ali and Wen-Wei Tang.

## Conflicts of interest

The authors declare no conflicts of interest.

## Supplementary Material

RA-015-D5RA00505A-s001
