# Supplementary material for: A new natural Cyperol A together with five known compounds from Cyperus rotundus L.: isolation, structure elucidation, DFT analysis, insecticidal and enzyme-inhibition activities and in silico study
Source: RSC Adv. 2025 Apr 11;15(15):11491–502. doi: 10.1039/d5ra00505a (PMC11987592; doi:10.1039/d5ra00505a)
Supplement: RA-015-D5RA00505A-s001 [file RA-015-D5RA00505A-s001.pdf]

Supplementary Information (SI) for RSC Advances.
This journal is © The Royal Society of Chemistry 2025

Supplementary materials

A new natural Cyperol A together with five known compounds from *Cyperus Rotundus L.*, isolation, structure elucidation, DFT analysis, insecticidal, enzyme inhibition activities and *in silico* study

Saqib Hussain Bangash, [ab] Muhammad Ibrahim,*[b] Akbar Ali,*[c] Chen-Yang Wei,[a] Amjad Hussain,[d] Moazama Riaz, [b] Fayyaz Ur Rehman,[e] Faiz Ahmed,[c] Rashad Al-Salahi [f] and Wen-Wei Tang*[a]

1. Guangxi Key Laboratory of Agri-Environment and Agric-Product Safety, College of Agriculture, Department of Crop Environment and Ecology, Guangxi university, Nanning, China.
2. Department of Applied Chemistry, Government College University Faisalabad, Pakistan,
3. Department of Chemistry, Government College University Faisalabad, Pakistan
4. Institute of Chemistry, University of Okaro, Okara-56300, Punjab, Pakistan.
5. Institute of Chemistry, University of Sargodha, Sargodha, Pakistan.

* Correspondence: wenweitg@163.com (WWT); *ibrahim@gcuf.edu.pk (MI); Tel. +86-15678178717

Table S1: The NBO analysis of **5a** at DFT/B3LYP/6-311G (d,p)

	Donor NBO (i)	Type	Acceptor NBO (j)	Type	E(2) kcal/mol	E(j)-E(i) a.u	F(i,j) a.u.
1.	C1 - C2	π	C3 - C4	π*	18.50	0.29	0.066
2.	C1 - C2	π	C5 - C16	π*	18.73	0.29	0.066
3.	C1 - C6	σ	C5 - H8	σ*	2.38	1.16	0.047
4.	C1 - C16	σ	C5 - C16	σ*	3.80	1.34	0.064
5.	C1 - O17	σ	C2 - C3	σ*	1.52	1.43	0.042
6.	C2 - C3	σ	C4 - O14	σ*	4.42	0.99	0.059
7.	C2 - O18	σ	C1 - C16	σ*	1.60	1.43	0.043
8.	C3 - C4	σ	C2 - O18	σ*	5.25	1.09	0.068
9.	C3 - C4	π	C1 - C2	π*	17.87	0.26	0.063
10.	C3 - C4	π	C5 - C16	π*	15.20	0.31	0.063
11.	C3 - H7	σ	C4 - C5	σ*	4.89	1.03	0.063
12.	C4 - C5	σ	C11 - C16	σ*	4.10	1.05	0.059
13.	C4 - O14	σ	C2 - C3	σ*	2.24	1.33	0.049
14.	C5 - H8	σ	C1 - C16	σ*	5.93	1.03	0.070
15.	C5 - C16	σ	C1 - O17	σ*	5.09	1.09	0.067
16.	C5 - C16	π	C1 - C2	π*	18.35	0.26	0.063
17.	C5 - C16	π	C3 - C4	π*	15.29	0.30	0.062
18.	C5 - C16	π	C11 - O12	π*	16.59	0.27	0.062
19.	C6 - H9	σ	C1 - O17	σ*	1.09	0.90	0.028
20.	C6 - H9	σ	C2 - O18	σ*	1.09	0.90	0.028
21.	C6 - H10	σ	C1 - O17	σ*	1.09	0.90	0.028
22.	C6 - H10	σ	C2 - O18	σ*	1.09	0.90	0.028
23.	C6 - O17	σ	C1 - C16	σ*	5.79	1.27	0.077
24.	C6 - O18	σ	C2 - C3	σ*	5.86	1.26	0.077
25.	C11 - O12	σ	C16	σ*	1.38	2.18	0.049
26.	C11 - O12	π	C5 - C16	π*	3.94	0.42	0.040
27.	C11 - H13	σ	C1 - C16	σ*	2.89	1.06	0.050
28.	C11 - C16	σ	C1 - C2	σ*	2.55	1.06	0.047

29.	O14 - H15	σ	C3 - C4	π*	3.15	0.75	0.048
30.	C1	σ	C2	σ*	1.25	10.99	0.105
31.	C2	σ	C3	σ*	1.42	11.01	0.112
32.	C3	σ	C4	σ*	2.27	11.07	0.142
33.	C4	σ	C4 - O14	σ*	2.00	10.41	0.129
34.	C5	σ	C16	σ*	2.20	11.18	0.140
35.	C6	σ	C6 - O18	σ*	0.95	10.41	0.089
36.	C11	σ	C16	π*	0.90	11.54	0.091
37.	O12	σ	C11	σ*	4.81	19.70	0.276
38.	O14	σ	C4	σ*	1.37	19.99	0.148
39.	C16	σ	C5	π*	1.75	10.83	0.123
40.	C16	σ	C1 - O17	σ*	1.75	10.42	0.121
41.	O17	σ	C1	σ*	1.80	19.87	0.169
42.	O18	σ	C2	σ*	1.91	19.85	0.174
43.	O12	σ	C11	σ*	11.52	1.50	0.118
44.	O12	π	C11 - H13	σ*	19.07	0.69	0.104
45.	O12	π	C11 - C16	σ*	20.39	0.65	0.104
46.	O14	σ	C3 - C4	σ*	5.94	1.12	0.073
47.	O14	π	C3 - C4	π*	5.58	0.44	0.048
48.	O14	π	C4 - C5	σ*	4.86	0.91	0.060
49.	O17	σ	C1 - C2	σ*	4.15	1.03	0.058
50.	O17	π	C1 - C2	π*	28.14	0.31	0.090
51.	O18	σ	C1 - C2	σ*	4.26	1.03	0.059
52.	O18	π	C1 - C2	π*	27.07	0.31	0.088

Table S2: Vibrational modes of **5a at** DFT/B3LYP/6-311G (d,p) level.

**Frequency**	**Intensity**	**Vibrational assignment (PED >5%)**
3814	118.77	*v*O14-H15(100%)
3208	2.00	*v*C3-H7(80%) *v*C5-H8(20%)
3207	3.99	*v*C3-H7(20%) *v*C5-H8(80%)
3115	48.48	*b*H6-C9-H10(100%)
3056	154.12	*v* H6-C9-H10(100%)
2928	202.68	*v*C11-H13(100%)
1768	445.11	*v*O12=C11(89%) C1-H13
1693	11.86	*v*C1=C2(11%) + *v*C4=C5(54%) + *v*C11-C16(11%)
1641	182.11	*v*C1=C16(11%) + *v*C4=C3(54%) + *v*C2-O18(11%)
1554	2.94	*b*H6-C9-H10 (90%)
1509	412.59	*v*C1=C2(-24%) + v_{as}O14-C4(-24%) + *b*H8-C5=C4=C16(-18%) + *b*C1=C2=C3(12%)
1500	105.77	*v*C5=C6(-14%) + *v*O7-C4(-14%) + *b*H15-C5=C6(16%) + *b*C1=C6=C5(18%) + *b*C4=C5=C6(18%)
1460	85.34	*v*C2=C3(-15%) + *v*C4=C5(-15%) + *b*H9-C8=O10(15%) + *τ*H17-C13-O12-C2(13%) + *τ*H18-C13-O12-C2(-23%)
1443	5.47	*v*C2=C3(45%) + *v*C4=C5(45%) + *τ*H18-C13-O12-C2(-15%)
1432	81.62	*b*H9-C8=O10(66%) + *τ*H18-C13-O12-C2(10%)
1322	241.00	*v*C1=C2(-65%) + *v*C6=C5(-65%) + *v*C6-C8(-65%) + *v*O7-C4(-65%)
1254	471.15	*b*H16-O7-C4(15%) + *b*H14-C3=C4(25%) + *b*H15-C5=C6(17%)
1230	51.00	*v*C1=C2(11%) + v_{as}O11-C1(11%) + *b*H16-O7-C4(14%) + *b*H14-C3=C4(-13%) + *b*H15-C5=C6(14%)
1204	0.41	*b*H17-C13-O12(-56%) + *τ*H18-C13-O12-C2(34%)
1200	167.29	*b*H16-O7-C4(-37%) + *b*H15-C5=C6(25%)
1164	298.38	*v*C6-C8(-45%) + v_{as}O11-C1(-45%) + *b*H14-C3=C4(-12%)
1136	14.70	*b*H17-C13-O12(41%) + *τ*H17-C13-O12-C2(36%) + *τ*C13-O11-C1=C2(-19%)

1096	110.85	vC6-C8(43%) + v_{as}O12-C2(43%) + bC5=C4-O7(-11%) + bC6=C1-O11(-11%)
1045	132.00	vC6-C8(13%) + v_{as}O7-C4(13%) + bC1=C2=C3(-14%) + b_{as}C3=C2-O12(-14%) + b_{as}C1-O11-C13(33%) + bC3=C2-O12(33%)
1030	0.03	τH9-C8-C6=C5(66%) + τ_{out}C8-C1=C5=C6(-14%) + τ_{as}C1=C6=C5=C4(-14%) + τC5=C6-C8=O10(-14%)
991	24.26	v_{as}C6-C8(-19%) + vO11-C13(-19%) + vO12-C2 (-19%) + bC3=C2-O12(19%) + bC1=C2=C3(19%) + bC1=C6=C5(19%) + b_{as}C4=C5=C6(19%)
953	96.27	v_{as}C6-C8(50%) + vO11-C13(50%) + vO12-C2 (50%) + b_{as}C1-O11-C13(15%) + bC3=C2-O12(15%)
845.77	0.0597	τH15-C5=C6-C8(-78%)
832	62.57	τH14-C3=C4=C5(70%) + τ_{out}O7-C3=C5=C4(14%) + τC1=C6=C5=C4(14%)
818	39.47	vC1=C2(53%) + vC1=C6(53%) + vO11-C1(53%)
760	69.99	vC1=C2(19%) + vO7-C4(19%) + bC5=C4-O7(19%) + b_{as}C6-C8=O10(19%) + bC3=C2-O12(-14%) + bC1=C2=C3(-14%) + bC1=C6=C5(-14%) + b_{as}C4=C5=C6(-14%)
735	60.98	b_{as}C1-O11-C13(-11%) + bC3=C2-O12(-11%) + bC1-O11-C13(50%) + bC3=C2-O12(50%) + bC6=C1-O11(50%) + b_{as}C6-C8=O10(50%)
702	0.42	τC2=C1=C6=C5(10%) + τ_{out}O11-C2=C6=C1(70%) + τ_{out}O12-C1=C3=C2(70%)
618	2.91	τH14-C3=C4=C5(-12%) + τ_{out}O7-C3=C5=C4(71%) + τ_{as}C1=C6=C5=C4(71%)
608	34.76	vC5=C6(-14%) + vO7-C4(-14%) + b_{as}C1=C6=C5(-46%) + b_{as}C6=C1-O11(-46%) + bC5=C4-O7(-46%)
513	0.57	vC1=C2(11%) + vC1=C6(11%) + bC1=C2=C3(-37%) + b_{as}C2=C3-O12(-37%)
494	3.78	τH9-C8-C6=C5(14%) + τ_{out}C8-C1=C5=C6(42%) + τC1=C6=C5=C4(42%) + τC5=C6-C8=O10(42%) + τ_{out}O7-C3=C5=C4(42%)
482	9.64	vC5=C6(16%) + vO7-C4(16%) + bC1=C6=C5(42%) + bC4=C5=C6(42%)
395	2.67	bC5=C4-O7(-41%) + bC6-C8=O10(-41%)
354	1.89	τ_{out}C8-C1=C5=C6(11%) + τC1=C6=C5=C4(11%) + τC5=C6-C8=O10(11%) + τ_{out}O7-C3=C5=C4(11%) + τ_{out}C8-C1=C5=C6(12%) + τC1=C6=C5=C4(12%) + τC5=C6-C8=O10(12%) + τ_{out}O7-C3=C5=C4(12%) + τC3=C2-C1-C6(56%) + τ_{out}O11-C2=C6=C1(56%)
313	6.08	bC5=C4-O7(50%) + bC5=C1-O11(50%)
290	167.12	τC3=C2=C1=C6(-70%) + τ_{out}O12-C1=C3=C2(-70%) + τC3=C2=C1=C6(-11%) + τ_{out}O11-C2=C6=C1(-11%)
267	5.94	τH16-O7-C4=C3(92%)
199	2.84	τH9-C8=C6=C5(13%) + τ_{out}C8-C1=C5=C6(53%) + τC1=C6=C5=C4(53%) + τC5=C6-C8=O10(53%)
156	7.76	bC1=C6=C5(-73%) + bC6=C1-O11(-73%) + bC5=C4-O7(-73%)
152	3.27	τC2=C1=C6=C5(55%) + τ_{out}C8=C1=C5=C6(10%) + τC1=C6=C5=C4(10%) + τC5=C6-C8=O10(10%) + τ_{out}O7-C3=C5=C4(10%)

93	2.37	τC2=C1=C6=C5(-15%) + τ_{out}C8-C1=C5=C6(68%) + + τC5=C6-C8=O10(68%)

v = stretching vibration, *b* = bending vibration, *τ* = rational vibrations

Table S3. Equations

$E^{(2)} = q_i \frac{(F_{i,j})^2}{\varepsilon_j - \varepsilon_i}$	*Equation 1*
$I = -E_{HOMO}$	*Equation 2*
$A = -E_{LUMO}$	*Equation 3*
$\eta = \frac{I - A}{2}$	*Equation 4*
$X = \frac{I + A}{2}$	*Equation 5*
$\mu = -\frac{I + A}{2}$	*Equation 6*
$\omega = \frac{\mu^2}{2\eta}$	*Equation 7*
$\sigma = \frac{1}{2\eta}$	*Equation 8*
$\mu_{total} = (\mu_{x^2} + \mu_{y^2} + \mu_{z^2})^{\frac{1}{2}}$	*Equation 9*
$\alpha_{mean} = \frac{1}{3}(\alpha_{xx} + \alpha_{yy} + \alpha_{zz})$	*Equation 10*
$\beta_{total} = [(\beta_{xxx} + \beta_{yyx} + \beta_{zzx})^2 + (\beta_{yyy} + \beta_{xxy} + \beta_{zzy})^2$	*Equation 11*

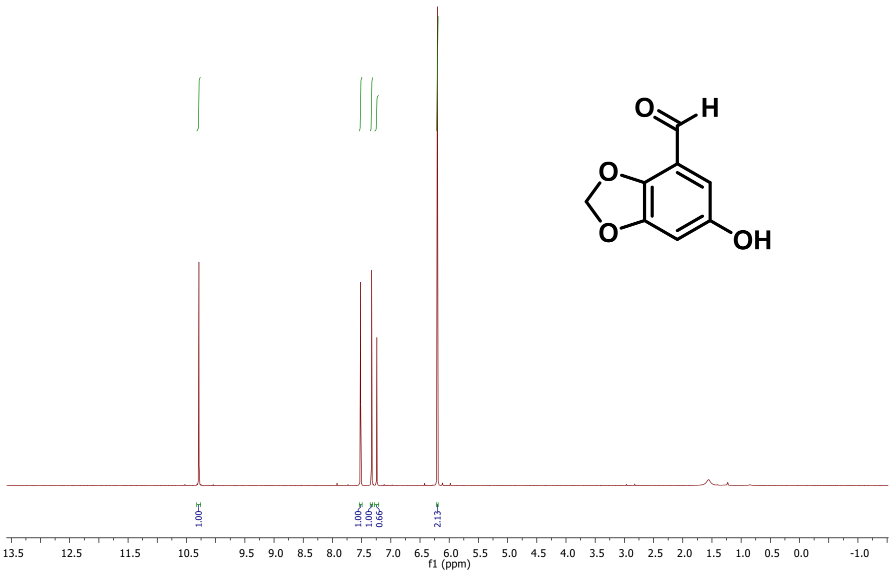

Figure S1:¹H-NMR data of the 6-hydroxybenzo[d][1,3]dioxole-4-carbaldehyde**.**

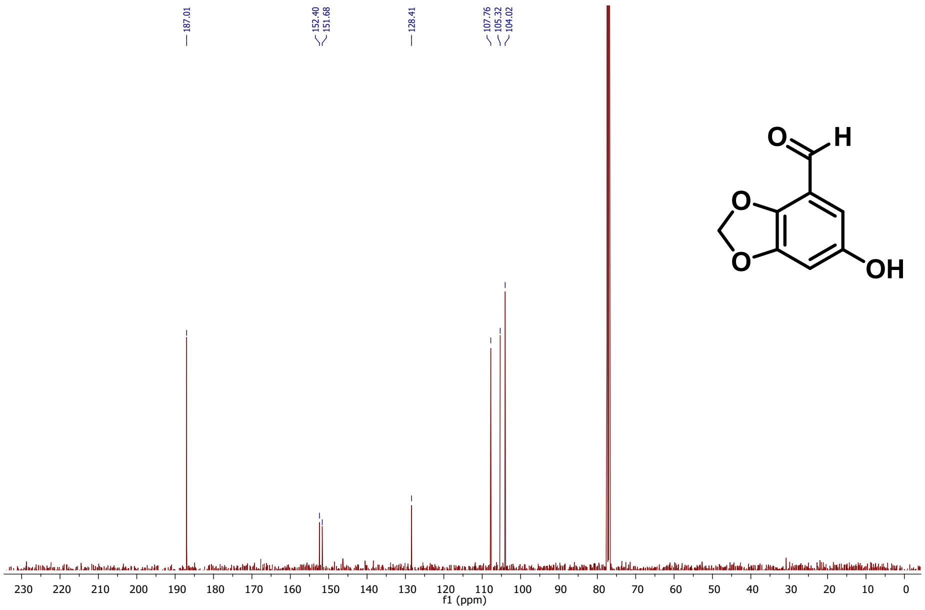

Figure S2: ¹³C-NMR data of the 6-hydroxybenzo[d][1,3]dioxole-4-carbaldehyde**.**

Instrument: JEOL 600H-1
Inlet: Direct Probe
Ionization mode: EI+
Run By: MASS LAB 104

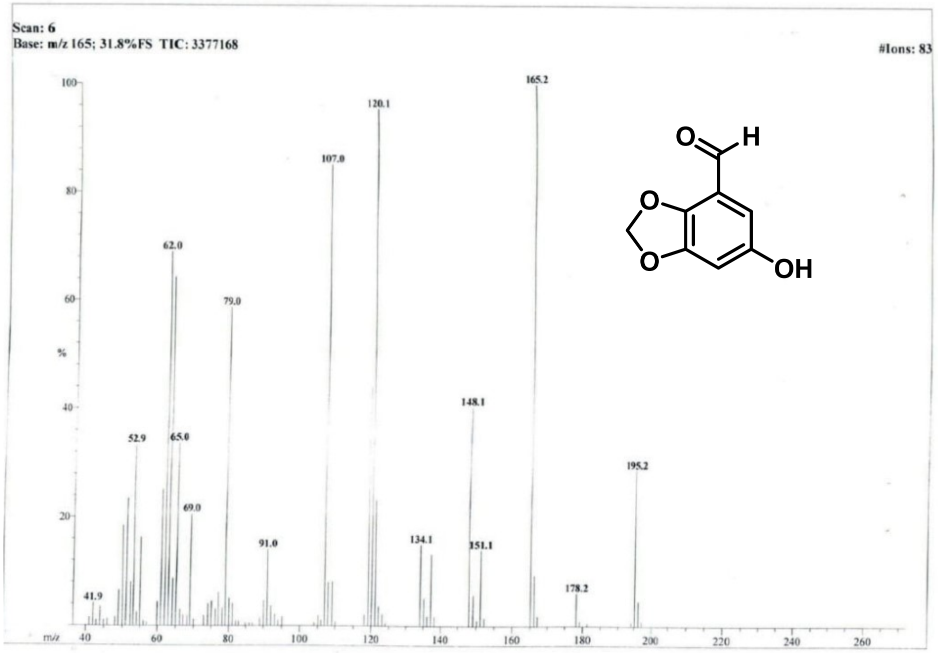

Figure S3: EIMS data of the 6-hydroxybenzo[d][1,3]dioxole-4-carbaldehyde.

Threshold: 1% of Base **Displayed TIC: 3377168**

Mass	%Base	Mass	%Base	Mass	%Base	Mass	%Base	Mass	%Base	Mass	%Base	Mass	%Base	Mass	%Base
40.9	1.5	51.0	23.5	64.0	8.7	75.0	4.5	91.0	14.1	109.1	8.2	136.1	1.9	166.2	9.4
41.9	4.2	52.0	8.0	65.0	33.7	76.0	3.1	92.0	3.7	118.1	2.2	137.1	13.3	167.2	1.9
43.0	1.1	52.9	33.3	66.0	3.0	77.0	6.1	93.0	2.2	119.1	44.0	138.1	1.7	178.2	6.0
43.9	3.5	54.0	2.5	67.0	2.0	78.0	3.3	94.0	1.1	120.1	95.4	148.1	40.4	195.2	28.8
44.9	1.1	55.0	16.3	68.0	1.9	79.0	58.7	95.0	1.7	121.1	23.2	149.1	5.7	196.2	4.6
45.9	1.2	60.0	4.4	69.0	20.6	80.0	5.1	105.1	2.0	122.1	3.6	150.1	1.1	197.2	1.0
47.9	1.6	61.0	25.2	70.0	1.2	81.0	4.2	106.1	1.1	123.1	2.3	151.1	14.0		
48.9	6.6	62.0	69.0	73.0	2.0	89.0	1.4	107.1	85.2	134.1	15.1	152.1	1.5		
50.0	18.5	63.0	64.3	74.0	4.1	90.0	4.7	108.1	8.1	135.1	5.1	165.2	100.0		

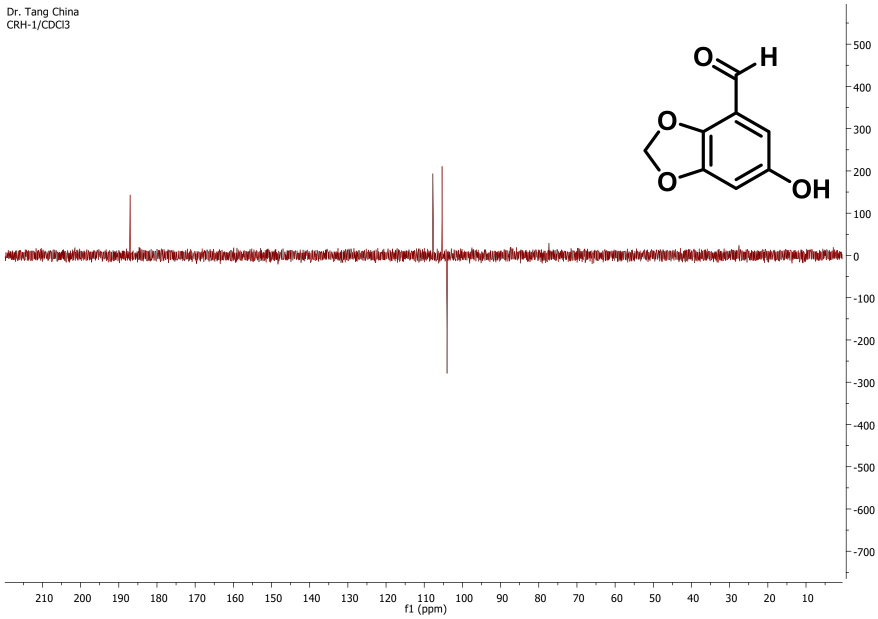

Figure S4: DEPT 135 ^{13}C-NMR data of the 6-hydroxybenzo[d][1,3]dioxole-4-carbaldehyde.

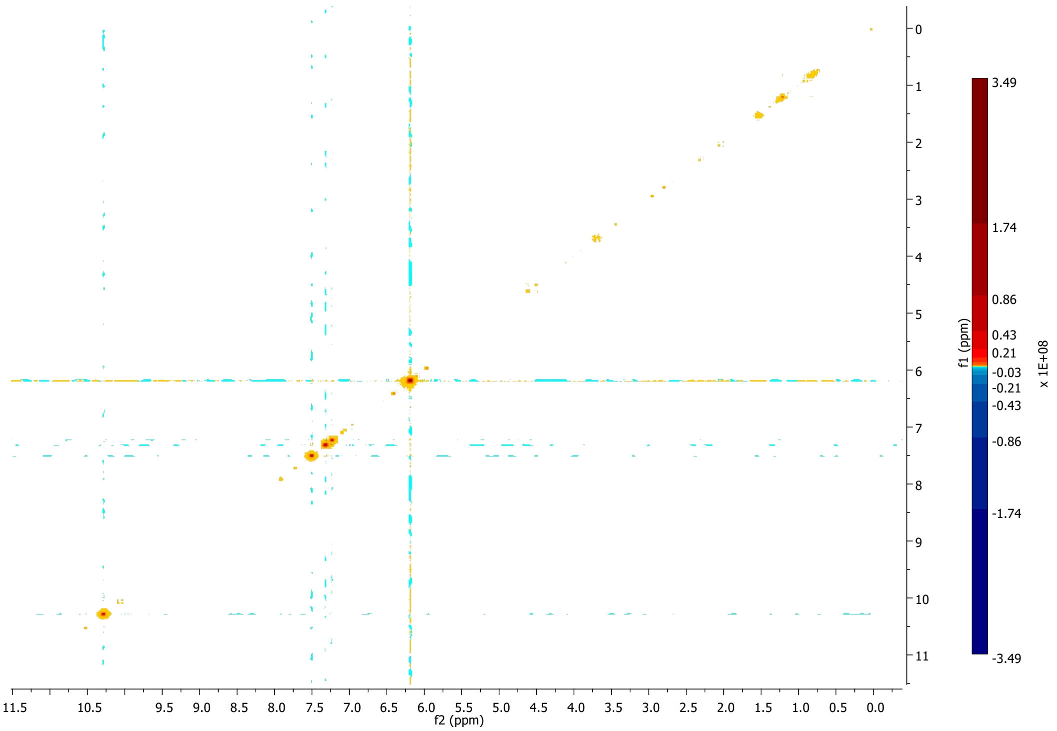

Figure S5: NOESY NMR Spectrum of the 6-hydroxybenzo[d][1,3]dioxole-4-carbaldehyde.

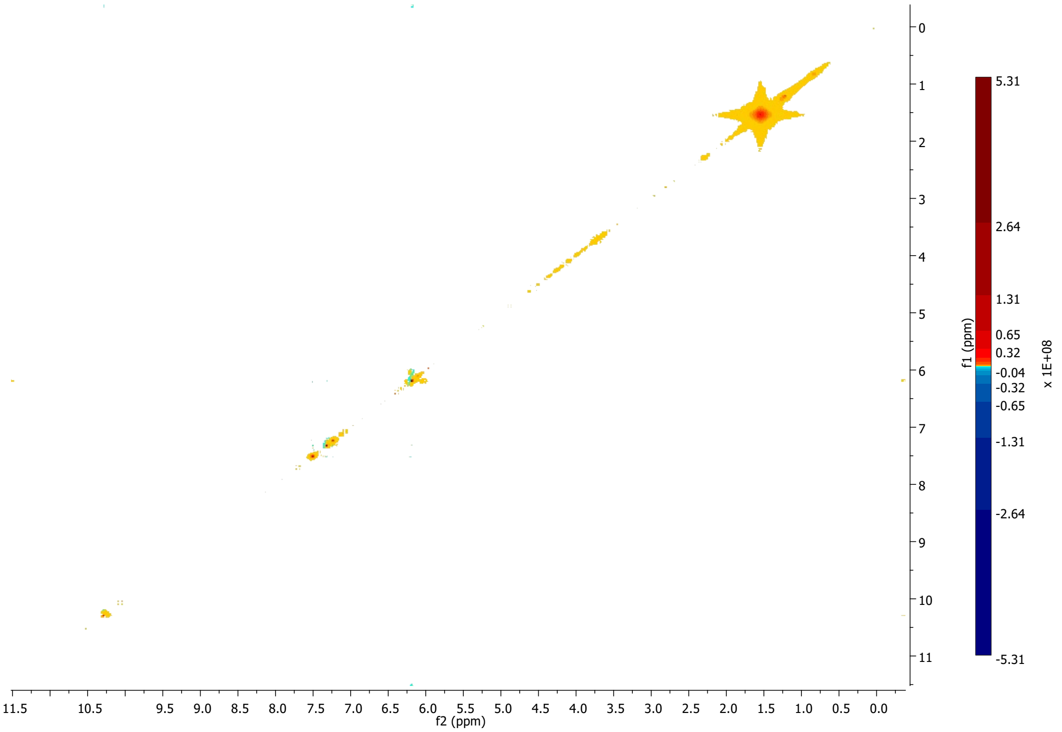

Figure S6: COSY NMR Spectrum of the 6-hydroxybenzo[d][1,3]dioxole-4-carbaldehyde.

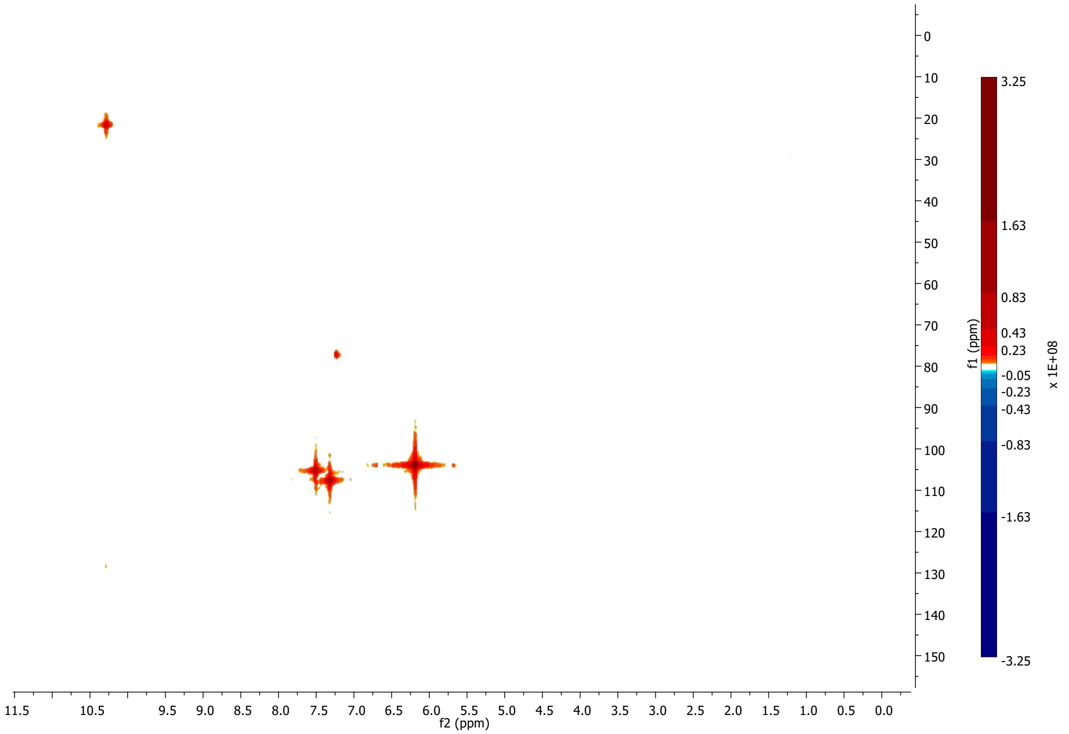

Figure S7: HSQC NMR spectrum of the 6-hydroxybenzo[d][1,3]dioxole-4-carbaldehyde.

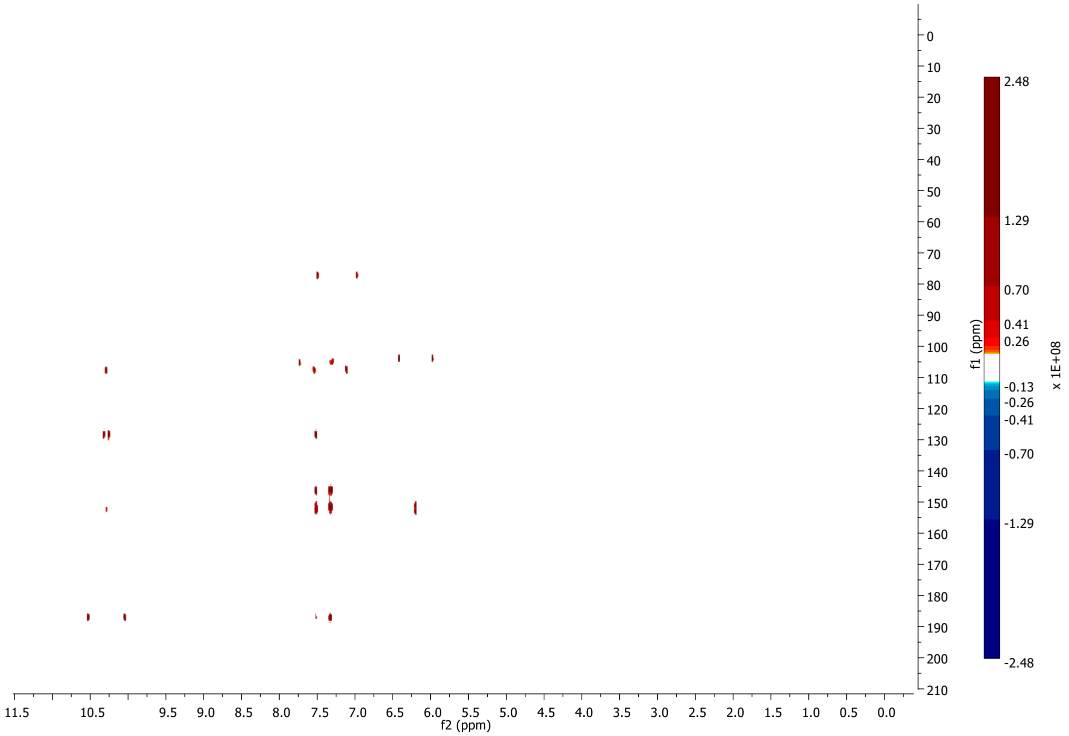

Figure S8: HMBC NMR spectrum of the 6-hydroxybenzo[d][1,3]dioxole-4-carbaldehyde.

Mass Spectrometry Conditions:

Ion source is heated electrospray (HR-ESIMS)，
Spray voltage of 3.0 kV in negative ion mode；
heater temperature: 350℃；
Capillary temp: 320℃，
Sheath gas flow rate: 10psi；
Aux gas flow rate: 2psi；
Scan type: Full MS
Scan range: 50~300m/z，
Resolution: 70,000

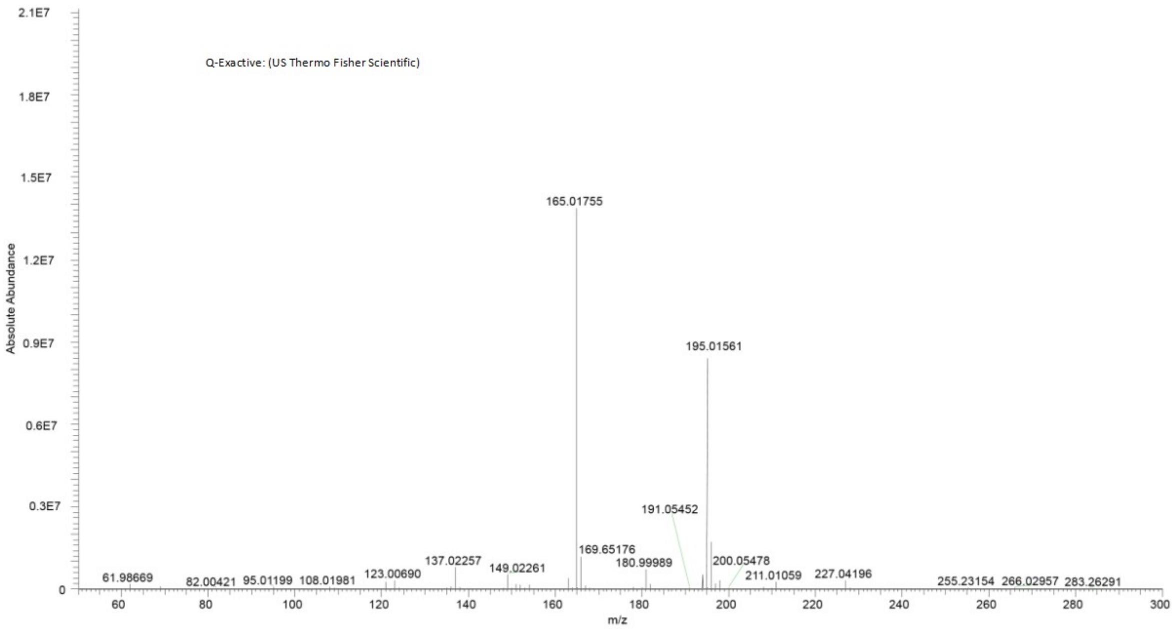

Figure S9: HR-ESIMS spectrum of the 6-hydroxybenzo[d][1,3]dioxole-4-carbaldehyde**.**
